# Self-reported strategy use in working memory tasks

**DOI:** 10.1038/s41598-024-54160-3

**Published:** 2024-02-28

**Authors:** Liisa Ritakallio, Daniel Fellman, Juha Salmi, Jussi Jylkkä, Matti Laine

**Affiliations:** 1 Department of Psychology, Åbo Akademi University, Turku, Finland; 2 Department of Neuroscience and Biomedical Engineering, Aalto University, Espoo, Finland; 3 Turku Brain and Mind Center, Turku, Finland

**Keywords:** Working memory, Strategy, Mnemonics, Task paradigm, Stimulus type, Psychology, Learning and memory, Working memory

## Abstract

Mnemonic strategies can facilitate working memory performance, but our knowledge on strategy use as a function of task characteristics remains limited. We examined self-reported strategy use in several working memory tasks with pretest data from two large-scale online training experiments. A three-level measure of strategy sophistication (no strategy, maintenance, manipulation) was coded based on participants’ open-ended strategy reports. A considerable portion of participants reported some memory strategy, and strategy sophistication was associated with objective task performance. We found a consistent effect of stimulus type: verbal stimuli (letters or digits) elicited higher strategy sophistication than nonverbal ones (colours or spatial positions). In contrast, the association between task paradigm and strategy sophistication was less consistent in the two experiments. The present results highlight the importance of self-generated strategies in understanding individual differences in working memory performance and the role of stimulus characteristics as one of the task-related determinants of strategy use.

## Introduction

Mnemonic strategies are well-known, age-old techniques for improving performance on specific memory tasks^1^. Given the experimental evidence that the use of mnemonics can facilitate memory performance^2,3^, strategy analysis is an important aspect of understanding what underlies an individual’s memory performance. In their classical paper “Human memory: A proposed system and its control processes”, Atkinson and Shiffrin^4^ emphasised that memory control processes such as rehearsal, coding, and search strategies are such a pervasive and integral part of human memory that any theory of memory in humans aiming at generality must take them into account. There is a sizable literature on strategy use in various memory tasks, including commonly used working memory (WM) tasks, but our knowledge of certain important aspects of spontaneous strategy use such as its dependence on task characteristics, is still quite limited. To address these issues, the present study analysed self-reported strategy use in a number of WM tasks, its relationship with objective task performance, and its variation as a function of stimulus type and paradigm. We attempted an internal replication by performing these analyses in two previously acquired datasets^5,6^.

Memory strategy is defined here as a self-generated, consciously used, verbalisable way of managing a WM task. Spontaneous strategy use in memory tasks reflects high-level executive control over encoding and recall^7–9^, aiming to facilitate performance. Strategy-related research can thus provide insights into the interactions between executive and memory functions and contribute to our knowledge on what memory tasks measure. Previous research has revealed a variety of self-generated strategies that are applied in WM tasks. Thus far, the most extensive study was conducted by Morrison et al.^10^. They examined self-reported strategy use of university students in seven WM tasks, including immediate and delayed serial recall, complex and running memory span, free recall, item recognition, and a missing item identification task. Their study concerned only a single type of stimuli, as all tasks except the last one employed words. Strategy use in each task was probed with multiple-choice questions. The results of Morrison et al.^10^ indicated substantial variation in participants’ strategy use both within and between tasks. Rehearsal and grouping were reported most often, followed by “concentrate”/no strategy, imagery, and use of semantics to aid memory. Distributions of self-generated WM strategies have been reported also in several other studies^6,11–19^. However, these distributions are challenging to compare because of differences in strategy queries (open-ended, multiple-choice), strategy coding systems, WM task paradigms, and stimuli. Thus, within-study analyses with a variety of WM tasks and a uniform strategy coding system are needed to examine the relationships between task characteristics, such as stimulus type and paradigm, and spontaneous strategy use.

Several studies have observed that certain types of spontaneously employed strategies are associated with higher WM task scores^6,11,13,15,19–23^. These mnemonics have been called as normatively effective^13^, elaborative^20^, or manipulation strategies^15^. The key characteristic of these strategies is that the WM contents are manipulated in one way or another by creating different associations or groupings of the memoranda to help remembering them. On the other hand, maintenance strategies (rehearsal/repetition) have shown much less or null advantage over no strategy^13,15,24^, or been a priori categorised as less effective or “passive” strategies together with no strategy use^11,13^. A recent review on this topic also highlighted the lack of efficacy of maintenance strategies.^10^.

Another fundamental issue on the relationship between strategy use and WM concerns the direction of the effect: do self-generated strategies help in compensating for WM limitations or does higher initial WM capacity enable their use? While research on spontaneous strategy use is correlative, there is causal evidence for the facilitatory role of strategies in WM performance from studies where participants were instructed to use a specific strategy^6,11,12,15–17,25–27^, but in some studies facilitation has been observed only in longer-term retrieval^28,29^. Recent meta-analytical evidence from WM training studies indicates positive effects of strategy instruction on actual performance^30^. All in all, previous research speaks for the relevance of strategies, especially those involving manipulation of the memoranda, in understanding WM performance.

It is reasonable to assume that WM task characteristics such as *the task paradigm* modulate spontaneous strategy use. For example, a spatial grouping and comparison strategy tailored for the n-back WM updating task^15^ would not be relevant for WM span tasks. In their study, Morrison et al.^10^ found considerable overlap in strategy use between their WM task paradigms. On all but the missing number identification task that was structurally and stimulus-wise disparate, rehearsal and grouping were the most common reported strategies. At the same time, strategy type distributions differed significantly between all WM tasks. The authors listed multiple potential reasons for these differences: serial order requirements, response demands, and updating/distraction modulate strategy choice. As the only systematic, more extensive study on the effects of WM task paradigm on spontaneous strategy employment is the one by Morrison et al.^10^, this issue is worth further investigation.

As all but one WM task employed words as stimuli, Morrison et al.^10^ could not probe the role of *the stimulus type* (verbal, visuospatial) in spontaneous strategy selection. This represents one key dimension on which theories of WM vary. Multicomponent models of WM, the most influential one presented by Baddeley and Hitch^31^, assume separate temporary stores for verbal versus visuospatial materials. Some models like the time-based resource-sharing model by Barrouillet and Camos^32^ postulate an independent rehearsal mechanism for verbal but not visuospatial materials. These models would readily account for differences in elaborating these types of memoranda. At the other end of this dimension are more unitary models such as the embedded-processes model by Cowan^33^ and the WM architecture by Oberauer^34^ that represent WM as heightened activation (attentional focus) of relevant information in long-term memory. In these models, and also in the interference model for complex span by Oberauer and colleagues^35^, seemingly modality-specific effects such as WM interference caused by a similar stimulus type are accounted for by overlap of featural dimensions without postulating separate phonological and visuospatial stores. Even in this theoretical context, elaborations of verbal versus visuospatial memoranda could vary as different features or feature clusters would provide different options for strategy use. A fundamental factor here may be language: it has been argued that “normal human cognition is language-augmented cognition”^36^, and verbal stimuli readily provide a number of ways to group and associate them phonologically and/or semantically to aid memory. In turn, remembering nonverbal materials would rely on visual/visuospatial processes and/or transformation into the language code (e.g., “upper right—middle–lower left” for a visuospatial WM task where target positions in a matrix need to be kept in mind). On the empirical side, an extreme example of how strategy effects may be tied to stimulus type is the seminal single-case study by Chase and Ericsson^37^. Over a two-year practice period, their participant developed a strategy that enabled him to have a digit span of nearly 80 items. Despite this, his letter span remained at 7 items, showing that his huge WM improvement was strictly tied to a self-developed complex strategy for memorising digits.

The short overview above underlines the importance of self-generated strategies in understanding individual performance differences and their underlying cognitive mechanisms in WM tasks. At the same time, it highlights the limits of our knowledge on the characteristics and determinants of strategy use in widely used WM task contexts. To address these knowledge gaps, the present study had three main aims. Our first aim was descriptive: we wanted to probe spontaneous strategy use with extensive WM batteries in order to characterise frequency and type of spontaneous strategy employment in various commonly used WM tasks. Second, we examined the relationships between strategy use and objective task performance. While this issue has been addressed in several earlier studies, it is important to confirm that in our study this association holds, as we are interested in the role of strategies in an individual’s WM performance. It also provides validity to the strategy coding system employed. Our third aim was more novel. By utilising factorial setups provided by our WM test batteries, we examined how stimulus type (digits, letters, colours, visuospatial patterns) and WM task paradigm (n-back, span, running memory, selective updating) modulate spontaneous strategy use. This should shed light on factors that govern the very wide inter- and intraindividual variation that characterises spontaneous strategy use in WM tasks. Our data stem from the pretests of two large-scale preregistered WM training experiments (https://osf.io/c9ygt^5^; https://aspredicted.org/r7qs9.pdf^6^).

As noted above, strategy coding systems vary between studies. The primary strategy measure in the present analyses, strategy sophistication, was developed and tested in our previous strategy-related study^6^. This measure is based on coding of participants’ open-ended strategy reports. Despite the need for independent raters and reliability analyses, we have preferred open-ended strategy questions, as our previous research indicates that they leave actual memory performance unaffected, in contrast to multiple-choice strategy queries that have been employed in some other WM-related strategy studies^7^. Following Fellman et al.^6^, the strategy sophistication variable is derived from the coded strategy types as a three-level continuous variable (no strategy, maintenance strategy, manipulation strategy). The use of this strategy variable is motivated by our previous studies that have linked strategy sophistication to actual memory performance^7^ and to practice-related performance improvement in WM training^6^. Similar patterns of results were also obtained with earlier analyses employing the primary strategy types instead of the strategy sophistication variable^15,19^.

## Experiment 1

### Methods

#### Participants

The anonymous participants (*n* = 200) of Experiment 1 were English-speaking adults in the age range of 18 to 50 years, recruited via the crowdwork website Prolific (http://www.prolific.co). Their average age was 32.12 years (*SD* = 8.27) and education length 16.4 years (*SD* = 3.36). There were 114 female and 86 male participants (no participant chose the category “other” in response to the gender question). The participants went through an extensive prescreening procedure to verify that they did not exhibit a psychiatric illness currently affecting their life, a neurological illness, a neurodevelopmental illness, CNS medication, drug use, heavy alcohol consumption on the night before, poor and uncorrected sight, a failed attention check, or intoxication at the time of the study.

The study was conducted in accordance with the Helsinki Declaration and approved by the Ethics Committee of the Departments of Psychology and Logopedics, Åbo Akademi University. Informed consent was obtained from all participants before enrolment. They were also informed of the contents of the study, the voluntary nature of participation, and the option to discontinue at any time without giving a reason. The participants received financial compensation (£50.52) for their participation in the whole training study, the pretest results of which are analysed here.

#### Tasks

The participants completed nine WM tasks representing four different paradigms: n-back tasks (with digits, letters, and colours), forward simple span tasks (with letters and colours), running memory tasks (with letters and colours), and selective updating tasks (with digits and colours). All tasks except n-back included practice trials before the actual tasks started. Task length varied between ca. 4–14 min, and task order was randomised. The participants also responded to questions on their strategy use, prior experience with the tasks, motivation, and alertness.

***N-Back with Digits (NBD)*** In this adaptive updating task^38^, digits from 1 to 9 are presented on the screen one at a time. The participant is to decide whether the currently presented item corresponds to the item presented n items back. The participant responds to each stimulus by pressing the designated “yes” or “no” button on the keyboard. The task included 12 blocks, consisting of n + 20 trials, six of which were targets and 14 non-targets. With the exception of the lowest 1-back level, the stimulus sequences included lures, i.e., stimuli appearing right before or after the target position that would match if they were in the target slot. Lures were used to minimise familiarity-based responding. The n-back levels 2- to 12-back contained four lures amongst the non-targets, two being presented just before and the other two just after a target position. The sequence of trials in a block proceeded in the following way: a blank screen for 450 ms, a stimulus displayed for 1500 ms, a blank screen for 450 ms, followed by the next stimulus. Task difficulty was adaptive: the participant started at the easiest level, 1-back, and was able to reach 12-back at the highest. If the participant obtained 15–17 trials correct, the level for the next block remained the same. If 18 or more trials were correct, the level was increased by one. If 14 or fewer trials were correct, the level was decreased by one. The dependent variable was the average level of n reached across the 12 blocks.

***N-Back with Letters (NBL)*** This task is the same as the NBD, the only difference being that the items are letters A to I instead of digits.

***N-Back with Colours (NBC)*** This task is the same as the NBD, the only difference being that the items are coloured squares (red, green, blue, yellow, black, purple, orange, pink, and grey) instead of digits.

***Forward Simple Span with Letters (FSSL)*** In this task that is based on the classic simple span paradigm^39^, letter sequences (ranging from A to I) varying in length are presented on the screen. The participant is not informed about the length of the coming sequence, but the task is always to recall the items in the order they are presented. The response is given after each sequence by clicking the correct letters in the correct order, on a row of horizontally aligned boxes with letters A-I shown on the screen. This task contained 6 trials with sequence lengths 4–9, presented in a randomised order (a sequence with 10 items was also included but not displayed due to a technical error). Each letter was shown for 1000 ms, and the inter-stimulus interval was 500 ms. The dependent variable was the total number of correctly recalled items in the correct order.

***Forward Simple Span with Colours (FSSC)*** This task is the same as the FSSL, the only difference being that the items are coloured squares (red, green, blue, yellow, black, purple, orange, pink, and grey) instead of letters, and that there were altogether 7 trials, one of each length 4–10.

***Running Memory with Letters (RML)*** This task is based on the paradigm presented by Pollack et al.^40^. Letter sequences ranging from A to I of varying length are presented on the screen. The length of each sequence is unknown to the participant, but the task is always to recall the last 4 items in the order they are presented. The participant responds after each sequence by clicking the correct letters in the correct order, on a row of horizontally aligned boxes with letters A-I shown on the screen. This task contained 8 trials with sequence lengths 4–11, presented in a randomised order. Stimulus exposure time was 1000 ms, with an inter-stimulus interval of 500 ms. The dependent variable was the total number of correctly recalled items in the correct order.

***Running Memory with Colours (RMC)*** This task is the same as the RML, the only difference being that the items are coloured squares (red, green, blue, yellow, black, purple, orange, pink, and grey) instead of letters.

***Selective Updating of Digits (SUD)*** This WM updating task is a slightly modified version of the original task by Murty et al.^41^. Five digits ranging from 0 to 9 are presented on the screen in a row of boxes. After 4000 ms, the digits are replaced by a blank screen for 100 ms, followed by a 2000 ms updating stage where a new row of boxes appears, with some containing new digits and others being blank. The task is to recall the final digit sequence, taking into account the updates. This task contained 20 trials, with the order of trials randomised for each participant. Half of the trials included only the initial sequence without any updates, while the other half included three updating stages. The dependent variable was the number of correctly recalled digits in the correct order on the updating trials.

***Selective Updating of Colours (SUC)*** This task is the same as the SUD, except that the items are coloured squares (red, green, blue, yellow, black, purple, orange, pink, and grey) instead of digits. Moreover, the stimulus display times were a little longer: the initial row of colours disappeared after 7000 ms, and the updating stage lasted for 5000 ms.

***Strategy use*** Upon completing each task, the participants were asked to describe their strategy for that task: ”Please describe in as much detail as possible what strategy (some memory technique to help you to remember) you used when performing this task.” The written responses were scored by two independent raters on two variables, namely primary strategy type and strategy level of detail. Only the first variable and its derivations were employed here, and thus the level of detail variable that correlated with strategy type is not discussed further. In the case that the participant described multiple strategies, only the first mentioned strategy was analysed. Strategy type was determined as: no strategy use (e.g. “I memorised all the items”), rehearsal/repetition (e.g. “I rehearsed the items in my mind”), grouping (e.g. “I grouped the letters in pairs”), updating (e.g. “I dropped the first digit when a new one appeared”), association (e.g. “I used the items to create a story”), selective focus (e.g. “I focused on remembering only a subset of the items and disregarded the rest”), other strategy (e.g. “I compared the new items with the old items”), or unspecified previous strategy (e.g. “I used the same strategy as in the previous task”). More details and further examples of these strategy types are presented in the coding instructions in the Supplementary Information, Appendix A. The scoring demonstrated very good inter-rater reliability, with the kappa coefficient for the strategy type varying between 0.84 and 0.94 for the different WM tasks. Our main dependent variable, strategy sophistication, was then derived from strategy type and formed a continuous variable with three levels in accordance with Fellman et al.^6^: (i) no strategy, (ii) maintenance strategy encompassing the strategy type rehearsal/repetition, and (iii) manipulation strategy involving the strategy types grouping, updating, association, and selective focus. Following Fellman et al.^6^, the maintenance strategy is conceived as active rehearsal of the presented stimuli in the order they were given. In turn, the more advanced manipulation strategies involve manipulation of the to-be-remembered WM contents in one way or another, such as stimulus grouping/chunking according to some principle, continuous updating of memoranda, creating associations for the stimuli, or focusing on only part of the stimuli.

***Prior experience, motivation, and alertness*** At the end of the session, the participants were asked about their motivation, alertness, and possible prior experience with any of the tasks they just completed. The questions were as follows: "How motivated were you to perform the tasks?"on the scale of 1 to 5 (1 = "Not at all motivated", 5 = Very motivated"). They were also asked: "How alert are you at the moment?" on the scale of 1 to 5 (1 = "Very tired", 5 = Very alert").

#### Procedure

The participants completed the study in a single test session (ca. 2 h 15 min) on our SOILE internet testing platform with their home computers or similar. In addition to the WM tasks and questionnaire items, the testing session included other cognitive tasks and questionnaires not reported here (for details, see Ritakallio et al.^5^).

#### Data preparation

Altogether 200 participants who fully completed the session without any cheating were preprocessed for possible exclusions. Cheating was defined as a yes-response to the question: “Did you use external tools (for example, writing, taking notes, or drawing) to help you solve the tasks? Please answer honestly, we guarantee that your response will not affect your payment. Your honest answer is critically important.” This question was presented at the very end of the pretest session. Those participants who were multivariate outliers at the 9 WM tasks (*n* = 1) were excluded from all analyses. Multivariate outliers were defined as scoring below the threshold of *p* < 0.001 in the Mahalanobis distance value (χ2 (9, 200) = 27.88^42^). Those participants who displayed unreliable effort (*n* = 0–3 per task) or were univariate outliers (*n* = 0) at any of the 9 WM tasks were excluded from analyses pertaining to the task in question, as were colour-blind participants (*n* = 5) from analyses concerning the colour task versions. Unreliable effort was present if a participant remained at the lowest level in the n-back tasks or did not recall any items correctly in the other tasks. Univariate outliers were defined as scoring three times the interquartile range below the 1st quartile or above the 3rd quartile, but none were found in the data. Finally, for analyses regarding strategy sophistication, those participants with strategy types *Other* or *Unspecified past strategy* were excluded. Please see Supplementary Information, Appendix B, Tables B.1 and B.2 for details on the final sample sizes per task.

#### Statistical approach

The data was analysed in the R Environment version 4.2.2 (R Core Team, 2017), using the “BayesFactor” package^43^ for computing the Bayes Factors (BFs). The interpretation of the BFs in this study followed the guidelines proposed by Kass and Raftery^44^, where BFs between 1 and 3 are defined as “weak evidence”, BFs between 3 and 20 as “positive evidence”, BFs between 20 and 150 as “strong evidence”, and BFs > 150 as “very strong evidence”. In each BF analysis, we used the default prior setting (Cauchy distribution scaling factor *r* = 0.707). Besides BFs, we also report estimates of between-group mean differences using a posterior distribution with 10 000 iterations coupled with their 95% credible intervals. The three questions we addressed were a description of self-reported strategy use in our battery of 9 WM tasks, the relationships between strategy use and objective task performance, and the variation of strategy use as a function of stimulus type and paradigm.

First, we conducted descriptive analyses of strategy sophistication (no strategy, maintenance strategy, or manipulation strategy). Moreover, we calculated the number of tasks where each participant employed a strategy.

Second, to assess potential associations between strategy use and task performance, we analysed strategy sophistication for each of the nine WM tasks. Associations between strategy use and task performance were assessed with Bayesian linear mixed effects (LME) models. As we were interested in the differences between each of the strategy group pairs (no strategy vs. maintenance strategy, no strategy vs. manipulation strategy, and maintenance vs. manipulation), we computed BFs between each pair. The dependent variable was task performance, strategy sophistication served as the fixed effect (treated as an ordinal variable: 0 = no strategy, 1 = maintenance, 2 = manipulation), and participant on the intercept served as the random factor.

Finally, to explore possible task-specific factors related to strategy use, we analysed strategy sophistication in connection to task paradigm (n-back vs. forward simple span vs. running memory; n-back vs. selective updating) and stimulus type (letters vs. colours; digits vs. colours). Possible task-specific factors were assessed with Bayesian LME models including the stimulus type * paradigm interaction. The data enabled us to construct two factorial models where stimulus type and paradigm were crossed: the first model consisted of the letter and colour variants of n-back, running memory and forward simple span, and the second one of the digit and colour variants of n-back and selective updating. Each analysis was specified in a separate model including the interaction between stimulus type and paradigm. The dependent variable was strategy sophistication (treated as an ordinal variable: 0 = no strategy, 1 = maintenance, 2 = manipulation), stimulus type and paradigm served as fixed effects, and participant on the intercept served as the random factor.

### Results

#### The first study aim: descriptive statistics on strategy employment in different WM tasks

**Figure 1:**
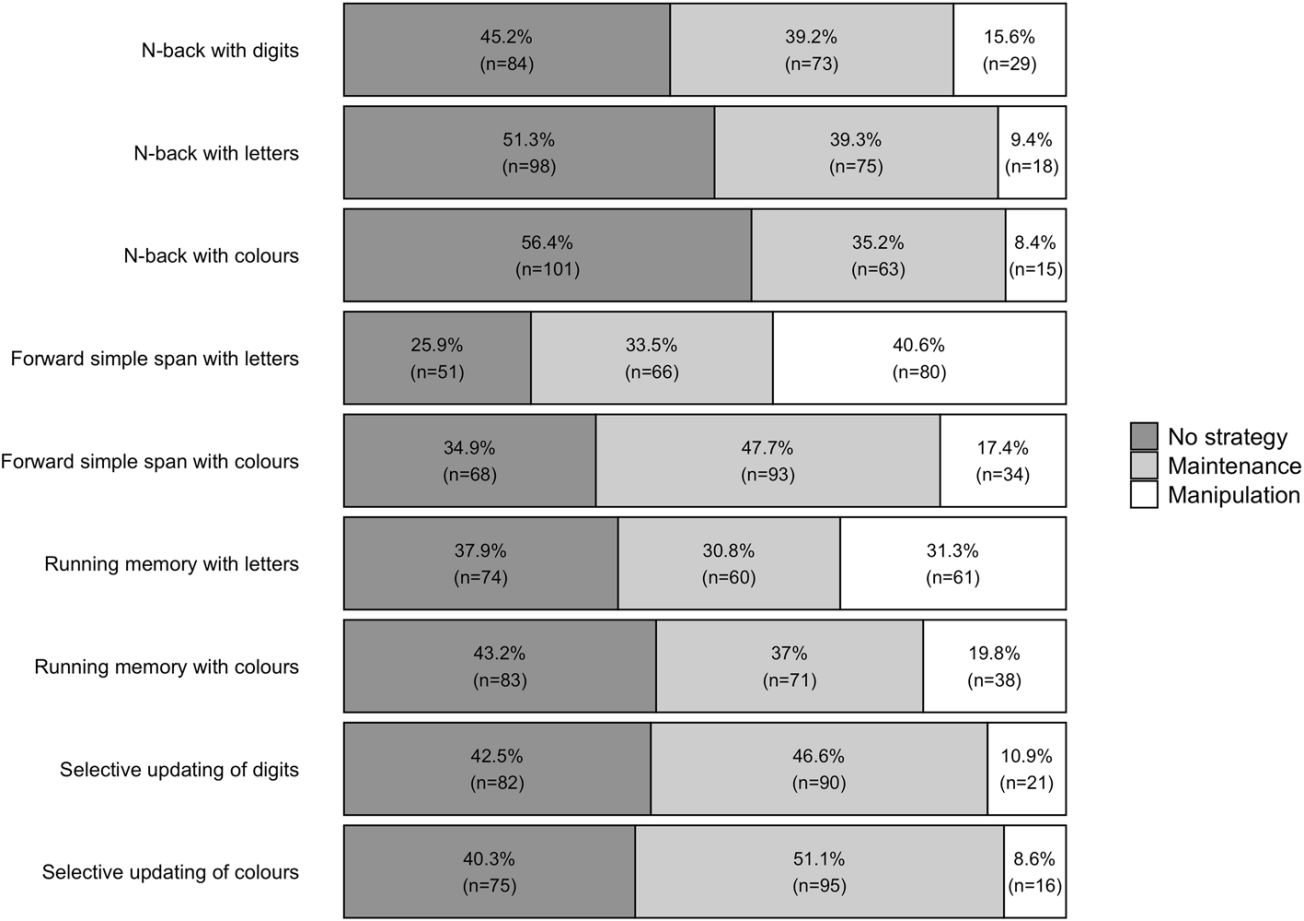
Task-by-task distribution of strategy use by the participants in Experiment 1. The bars depict the percentage of participants using either no strategy, a maintenance strategy, or a manipulation strategy. Those participants that had missing data for any of the tasks or whose strategy type for any of the tasks was either other strategy or unspecified past strategy, were excluded from this analysis.

**Figure 2:**
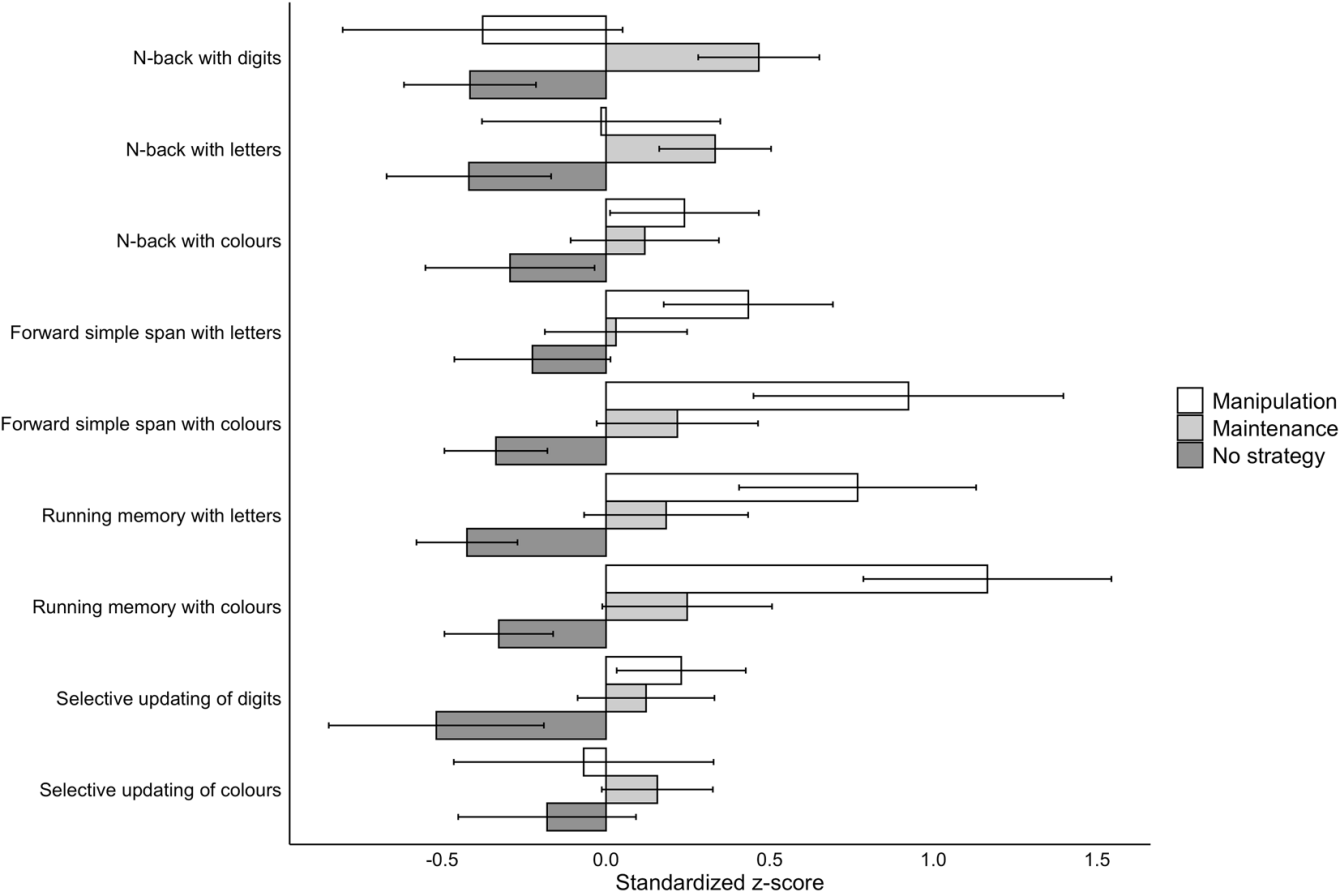
Illustration of task performance grouped by task and strategy use in Experiment 1. Task performance values are standardised using z-scores, providing a comparable metric across different tasks. Whiskers extending from the bars represent 95% confidence intervals. Those participants that had missing data for any of the tasks or whose strategy type for any of the tasks was either other strategy or unspecified past strategy, were excluded from this analysis.

The percentage of participants that used a strategy varied between 43.58 and 74.11%, depending on the task (see Figs. 1, 2; see also Supplementary Information, Appendix C, Tables C.1–C.2 for details). Strategy use was most common in the span tasks and least common in the n-back tasks. A maintenance strategy (i.e., rehearsal/repetition) was chosen by 30.77–51.08% of the participants. A manipulation strategy (i.e., grouping, updating, association, or selective focus), on the other hand, was chosen by 8.38–40.61% of the participants.

The number of tasks in which the participants used a strategy varied between 0 and 9. On average, participants employed a strategy (either maintenance or manipulation) in 8.68 of the 9 tasks (*SD* = 8.43), and the most common number of tasks to employ strategies in was nine, i.e. all tasks. A maintenance strategy was used on average in 6.08 tasks (*SD* = 3.38) and most commonly in three tasks (when used at all). A manipulation strategy, on the other hand, was used on average in 3.25 tasks (*SD* = 2.01) and most commonly in one task (when used at all).

#### The second study aim: relationships between strategy use and objective task performance

The participants’ strategy use was examined in connection to their task performance (see Table 1 for details). For this purpose, we ran pairwise Bayesian LMEs for each of the WM tasks. Those participants who employed any strategy (either maintenance or manipulation) performed better than no strategy users in all n-back tasks as well as in the FSSL. Maintenance strategy users also displayed better performance on the selective updating tasks when compared to the participants who did not use a strategy, and weak evidence for a similar difference was found in the case of the FSSC and the RML. On the other hand, there was weak evidence against a performance difference in the RMC when comparing maintenance strategy users to those who did not use a strategy. Manipulation strategy users also displayed better performance on the running memory tasks when compared to the participants who did not use a strategy. On the other hand, there was weak to positive evidence against a performance difference in the selective updating tasks and the FSSC when comparing manipulation strategy users to those who did not use a strategy.

**Table 1:** Parameter estimates for the Bayesian LMEs on strategy use and task performance.

Task	No strategy versus Maintenance		No strategy versus Manipulation		Maintenance versus Manipulation	
	M_diff_ [95% HDI]^a^	BF_H1_ ± error	M_diff_ [95% HDI]^a^	BF_H1_ ± error	M_diff_ [95% HDI]^b^	BF_H1_ ± error
NBD	− 0.28 [− 0.41, − 0.14]	**> 150 ± 0**	− 0.55 [− 0.71, − 0.4]	**> 150 ± 0**	− 0.26 [− 0.46, − 0.05]	**3.27 ± 0.01**
NBL	− 0.22 [− 0.33, − 0.1]	**> 150 ± 0**	− 0.5 [− 0.67, − 0.33]	**> 150 ± 0**	− 0.26 [− 0.49, − 0.05]	**3.56 ± 0.01**
NBC	− 0.21 [− 0.31, − 0.1]	**140.84 ± 0**	− 0.54 [− 0.72, − 0.38]	**> 150 ± 0**	− 0.31 [− 0.52, − 0.11]	**20.12 ± 0**
FSSL	− 2.4 [− 3.81, − 0.99]	**30.34 ± 0**	− 2.82 [− 4.14, − 1.34]	**> 150 ± 0**	− 0.4 [− 1.49, 0.7]	1/5.88 ± 0.08
FSSC	− 1.56 [− 3.02, − 0.14]	1.22 ± 0.02	− 0.51 [− 2.65, 1.67]	1/5.56 ± 0.06	1.02 [− 0.71, 2.67]	1/3.23 ± 0.04
RML	− 1.05 [− 1.96, − 0.16]	1.73 ± 0.01	− 1.36 [− 2.29, − 0.45]	**9.29 ± 0**	− 0.31 [− 1.08, 0.57]	1/5.56 ± 0.07
RMC	0.73 [− 1.68, 0.22]	1/2.22 ± 0.04	− 1.89 [− 2.98, − 0.63]	**24.02 ± 0**	− 1.15 [− 2.19, − 0.14]	1.77 ± 0.01
SUD	− 4.05 [− 5.3, − 2.7]	**> 150 ± 0**	− 0.16 [− 2.28, 1.7]	1/5.26 ± 0.04	3.72 [1.66, 5.65]	**134.78 ± 0**
SUC	− 3.79 [− 5.31, − 2.32]	**> 150 ± 0**	− 1.86 [− 4.8, 0.77]	1/2 ± 0.02	1.62 [− 0.51, 3.72]	1/1.64 ± 0.02

Bolded values indicate Bayes Factors (BFs) of 3 or greater. Estimates are from 10 000 samples of the posterior distribution; Mdiff = mean group differences; HDI = highest density interval. NBD = N-back with digits; NBL = N-back with letters; NBC = N-back with colours; FSSL = Forward simple span with letters; FSSC = Forward simple span with colours; RML = Running memory with letters; RMC = Running memory with colours; SUD = Selective updating of digits; SUC = Selective updating of colours.

^a^Positive values represent greater performance with no strategy.

^b^Positive values represent greater performance with a maintenance strategy.

When comparing the two strategy sophistication levels, maintenance and manipulation, against each other, those who used a manipulation strategy fared better at the n-back tasks, while those who used a maintenance strategy fared better at SUD. However, on the majority of the tasks there was either positive evidence against a difference between the use of these two strategies or merely weak evidence for or against a difference.

#### The third study aim: how stimulus type and WM task paradigm modulate spontaneous strategy use

**Figure 3:**
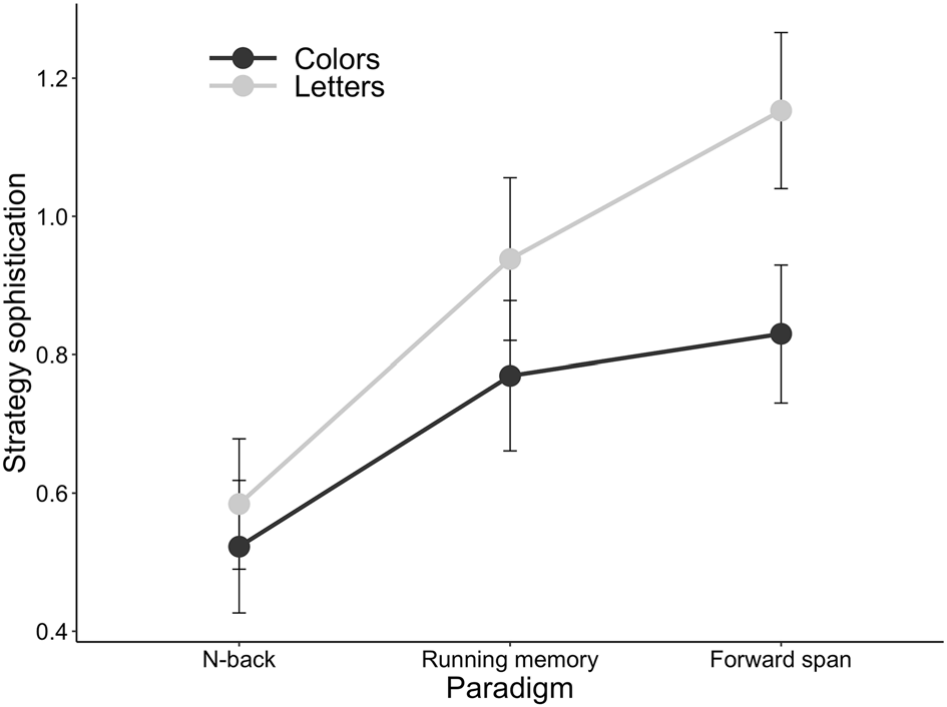
Strategy use by task paradigm and stimulus type in the first Bayesian LME analysis in Experiment 1. The y axis displays the mean of strategy sophistication (strategy was treated as an ordinal scale variable with the following values: 0 = No strategy, 1 = Maintenance, 2 = Manipulation). The x axis depicts the task paradigm.

The participants’ strategy use was explored in connection to two task characteristics, paradigm and stimulus type (see Table 2 and Fig. 3 for details). To analyse the associations between task paradigm, stimulus type and strategy sophistication, we ran two Bayesian LMEs including the stimulus type * paradigm interaction. Thus, these two analyses covered the factorial setups crossing stimulus type and paradigm that were possible in the present data. The analyses included strategy sophistication as the dependent variable, stimulus type and task paradigm as fixed effects, and participant as the random factor.

**Table 2:** Parameter estimates for the Bayesian LMEs on strategy sophistication and task characteristics.

	Analysis A			Analysis B		
Model	Comparison	M_diff_ [95% HDI]^a^	BF_H1_ ± error	Comparison	M_diff_ [95% HDI]^a^	BF_H1_ ± error
Stimulus	Letters versus Colours	0.18 [0.15, 0.22]	**> 150** ± 1.58 %	Digits versus Colours	− 0.09 [0.05, 0.13]	1/1.64 ± 2.36 %
Paradigm			**> 150** ± 1.79 %	N-back versus Selective updating	− 0.07 [− 0.11, − 0.03]	1/4.55 ± 2.77 %
	N-back versus Running memory	− 0.29 [− 0.34, − 0.24]				
	N-back versus Forward simple span	− 0.43 [− 0.48, − 0.38]				
	Running memory versus Forward simple span	− 0.14 [− 0.19, − 0.09]				
Stimulus * Paradigm	Interaction	NA^b^	1.15 ± 2.11 %		NA^b^	1/1.45 ± 3.08 %

Bolded values indicate Bayes Factors (BFs) of 3 or greater. Estimates are from 10 000 samples of the posterior distribution. Those participants that had missing data or whose strategy type was either other strategy or unspecified past strategy for any of the tasks within a given analysis were excluded from that analysis.

^a^Positive values represent higher strategy sophistication with the first mentioned stimulus type or paradigm.

^b^Too many comparisons.

The first analysis (see Fig. 3) included two stimulus types (letters, colours) and three WM paradigms (n-back, running memory, forward simple span) as fixed effects. We observed very strong evidence for the effect of stimulus as well as for the effect of paradigm. Merely weak evidence for the stimulus * paradigm interaction was detected.

The second analysis included two stimulus types (digits, colours), and two WM paradigms (n-back, selective updating) as fixed effects. We observed positive evidence against the effect of paradigm. Weak evidence against the effect of stimulus as well as against the stimulus * paradigm interaction was detected.

### Discussion

Self-reported strategy use was quite common as for any of the WM tasks, with about half to two-thirds of the participants employing some strategy. Broadly speaking, these frequency distributions between no strategy (a common choice in any WM task here), maintenance (the most frequent strategy choice), and manipulation (a less frequent choice) are in line with previous related studies^10,15,18,19^. At the same time, variability in strategy use between the tasks was evident, enabling us to look into the effects of task paradigm and stimulus type on strategy employment.

The results of Experiment 1 provided strong support for the importance of strategies in WM performance. As depicted in Table 1, in eight out of nine WM tasks there was positive to very strong evidence for a performance difference in favour of strategy users as compared to the participants who did not use a strategy. The exception was the FSSC, where the evidence for a similar effect was only weak. Moreover, positive or strong evidence for a difference between maintenance versus manipulation was obtained for four WM tasks (all three n-back tasks and the SUD). In the case of SUD, maintenance strategy users exhibited better performance, while the results for n-back were in the expected direction of a better performance when employing a manipulation strategy. These results are in line with several previous studies^6,11,13,15,19^.

Using the factorial setup provided by our WM test battery, the first analysis with n-back, running memory and forward simple span tasks provided evidence for an association between stimulus type and strategy sophistication as well as between WM paradigm and strategy sophistication. Strategy sophistication was higher with letters than with colours as stimuli and increased when moving from n-back to running memory and forward simple span tasks. However, these main effects of stimulus type and task paradigm were absent when we compared digits versus colours on n-back versus selective updating. Besides low overall strategy sophistication values, a possible reason for this result is that irrespective of stimulus type, it may be difficult to find another functional strategy than maintenance (rehearsal/repetition) for selective updating. In line with this interpretation, Figs. 1, 2 show that the percentage of maintenance strategy users was highest for the selective updating tasks. Performance-wise, stimulus type had an effect even for selective updating: those employing a maintenance strategy were superior to manipulation strategy users when the stimuli were digits, but not when they were colours (Table 1).

## Experiment 2

We attempted to replicate and extend the results of Experiment 1 by running similar analyses with another large data set where a somewhat different WM battery had been employed. This was deemed as important for ensuring the robustness of the main findings.

### Methods

#### Participants

The anonymous participants (*n* = 296) of this study were English-speaking adults in the age range of 18–50 years, recruited via the crowdwork website Prolific (http://www.prolific.co). Their average age was 34.0 years (SD = 8.44) and education length 15.8 years (SD = 3.48). There were 183 female and 107 male participants (no one chose the category ”other” in response to the gender question). The participants went through an extensive prescreening procedure to verify that they did not exhibit a psychiatric illness currently affecting their life, a neurological illness, a neurodevelopmental illness, CNS medication, drug use, heavy alcohol consumption on the night before, poor and uncorrected sight, a failed attention check, or intoxication at the time of the study.

The study was conducted in accordance with the Helsinki Declaration and approved by the Ethics Committee of the Departments of Psychology and Logopedics, Åbo Akademi University. Informed consent was obtained from all participants before enrolment. The participants were also informed of the contents of the study, the voluntary nature of participation, and the option to discontinue at any time without giving a reason. The participants received financial compensation (£10.00) for their participation in the whole training study, the pretest results of which are analysed here.

#### Tasks

The participants completed 10 WM tasks representing four different paradigms: n-back tasks (with digits, letters, colours, and boxes), forward and backward simple span tasks (with digits and boxes) and running memory tasks (with digits and boxes). All tasks except n-back included practice trials before the actual tasks started. Task length varied between ca. 6–14 min and task order was randomised, with the exception that each backward simple span task was always presented after the forward simple span task with the same stimulus type (i.e., forward simple span with digits was always followed by backward span with digits and forward simple span with boxes was always followed by backward span with boxes). As with Experiment 1, the participants also responded to questions on their strategy use, prior experience with the tasks, motivation, and alertness.

***N-Back with Digits (NBD)*** This is the same as the NBD in Experiment 1.

***N-Back with Letters (NBL)*** This is the same as the NBL in Experiment 1.

***N-Back with Colours (NBC)*** This is the same as the NBC in Experiment 1.

***N-Back with Boxes (NBB)*** This task is the same as the NBD, the only difference being that the items are flashing squares on a grid (9 × 9) instead of digits.

***Forward Simple Span with Digits (FSSD)*** This is the same as the FSSD in Experiment 1.

***Forward Simple Span with Boxes (FSSB)*** This task is the same as the FSSD, the only difference being that the items are flashing squares on a grid (9 × 9) instead of digits.

***Backward Simple Span with Digits (BSSD)*** This is the same as the FSSD, the only difference being that the digits need to be recalled in the opposite order, from last to first.

***Backward Simple Span with Boxes (BSSB)*** This task is the same as the BSSD, the only difference being that the items are flashing squares on a grid (9 × 9) instead of digits.

***Running Memory with Digits (RMD)*** This is the same as the RML in Experiment 1, the only difference being that the items are digits 1–9 instead of letters.

***Running Memory with Boxes (RMB)*** This is the same as the RMD, the only difference being that the items are flashing squares on a grid (9 × 9) instead of digits.

***Strategy use*** Upon completing all tasks, the participants were asked to describe their strategy for each task: “Please describe in as much detail as possible what strategy (some memory technique to help you to remember) you used when performing this task.” The written responses were scored by two independent raters on three variables, namely primary strategy type, strategy level of detail, and number of strategies. Only the first variable and its derivations were employed here, and thus the level of detail variable that correlated with strategy type is not discussed further. Also here, in the case that the participant described multiple strategies, only the first mentioned strategy was analysed. Strategy type was determined as: no strategy use (e.g. “I pressed the N-key if the current white box was the same as the white box presented before it, or the M-key if it was not the same”), rehearsal (e.g. “I repeated the digits silently in my mind”), visuo-motor rehearsal (e.g. “I counted the digits with my fingers”), grouping (e.g. “I created groups of 3 digits”), updating (e.g. “I created a group of digits in my mind and dropped the last digit when a new digit appeared”), grouping and comparison (e.g. “I split the digits into different series, and compared those to each other”), transformation (e.g. “I converted the locations into numbers”), semantics (e.g. “I created words from the letters (e.g., C–R–S = Corn–Rose–Sand”), phonology (e.g. “I tried to make syllables out of the letters”), imagery (e.g. “I tried to visualise the letters as snakes”), visualisation (e.g. “I tried to visualise the letter sequence in my mind”), guessing (e.g. “I just used intuition”), familiarity (e.g. “I recalled the digits that were familiar”), or other strategy (e.g. “I tried to keep all the digits in my mind”). More details and further examples of these strategy types are presented in the coding instructions in the Supplementary Information, Appendix D. The scoring demonstrated good inter-rater reliability, with the kappa coefficient for the strategy type varying between 0.66 and 0.96 for the different WM tasks. Our main dependent variable, strategy sophistication, was then derived from strategy type and formed a continuous variable with three levels as in Fellman et al.^6^: (i) no strategy, (ii) maintenance strategy encompassing the strategy types rehearsal, visuo-motor rehearsal, and familiarity, and (iii) manipulation strategy involving the strategy types grouping, updating, grouping and comparison, transformation, semantics, phonology, imagery, and visualisation. Following Fellman et al.^6^, the maintenance strategy is conceived as active rehearsal of the presented stimuli in the order they were given. In turn, the more advanced manipulation strategies involve also manipulation of the to-be-remembered WM contents in one way or another, such as stimulus grouping/chunking according to some principle, continuous updating of memoranda, or creating associations for the stimuli.

There were two differences regarding strategy information gathering and coding in comparison to between Experiment 1 and Experiment 2. Firstly, there were some differences in the strategy types to be coded. Both experiments included the strategy types no strategy, rehearsal (in Experiment 1 called rehearsal/repetition), grouping, updating, and other strategy. Only Experiment 1 contained association and selective focus, while only Experiment 2 contained visuo-motor rehearsal, grouping and comparison, transformation, semantics, phonology, imagery, visualisation, guessing, and familiarity. Some of the strategy types unique to one experiment were partly overlapping with one or more types unique to the other experiment. Secondly, the strategy information was gathered at different points in the experiments. In Experiment 1, the participants were asked about their strategy for each task upon completing the task in question, while in Experiment 2, the participants were asked about their strategy for each task upon completing all the tasks.

***Prior experience, motivation, and alertness*** At the end of the pretest session, the participants were asked about their motivation, alertness, and possible prior experience with any of the tasks they just completed. The questions were as follows: "How motivated were you to perform the tasks?" assessed on the scale of 1–5 (1 = "Not at all motivated", 5 = Very motivated"). They were also asked: "How alert are you at the moment?" assessed on the scale of 1 to 5 (1 = "Very tired", 5 = Very alert").

#### Procedure

The participants completed the study in a single test session (ca. 2 h) on our SOILE internet testing platform employing their home computers or similar. In addition to the aforementioned tasks and questionnaire items, the testing session included other cognitive tasks and questionnaires not reported here (for details, see Fellman et al.^6^).

#### Data preparation

The data was prepared with the same procedure as with Experiment 1. Cheating, unreliable effort, and univariate outliers were also defined in a similar manner, while multivariate outliers in this instance were defined as scoring below the threshold of *p* < 0.001 in the Mahalanobis distance value (χ2 (10, 296) = 29.59^42^). Altogether 296 participants were preprocessed for possible exclusions, followed by the exclusion of multivariate outliers (*n* = 6) as well as taskwise those with unreliable effort (*n* = 0–11 per task), univariate outliers (*n* = 0) and colour-blind participants (*n* = 2). Finally, for analyses regarding strategy sophistication, those participants with strategy types *Other* or *Unspecified past strategy* were excluded. Please see Supplementary Information, Appendix E, Tables E.1 and E.2 for details on the final sample sizes per task.

#### Statistical approach

The data was analysed with the same procedure as with Experiment 1. For assessing potential associations between strategy use and task performance, each of the ten WM tasks was analysed. For exploring possible task-specific factors behind strategy use, the task paradigm comparisons were n-back versus backward span versus forward span versus running memory, and the stimulus type comparisons were digits versus boxes.

### Results

#### The first study aim: descriptive statistics on strategy employment in different WM tasks

**Figure 4:**
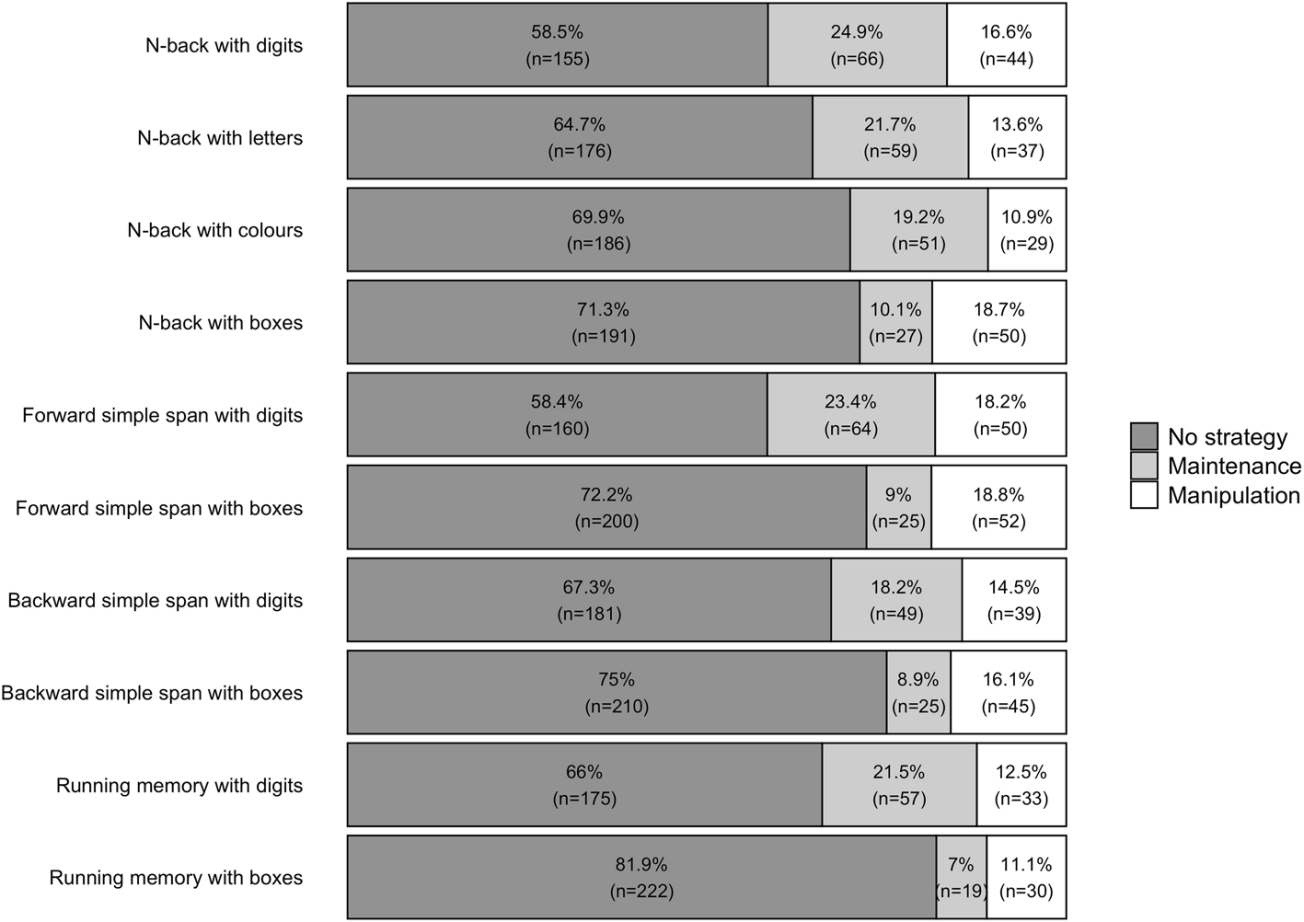
Task-by-task distribution of strategy use by the participants in Experiment 2. The bars depict the percentage of participants using either no strategy, a maintenance strategy, or a manipulation strategy. Those participants that had missing data for any of the tasks or whose strategy type for any of the tasks was other strategy, were excluded from this analysis.

**Figure 5:**
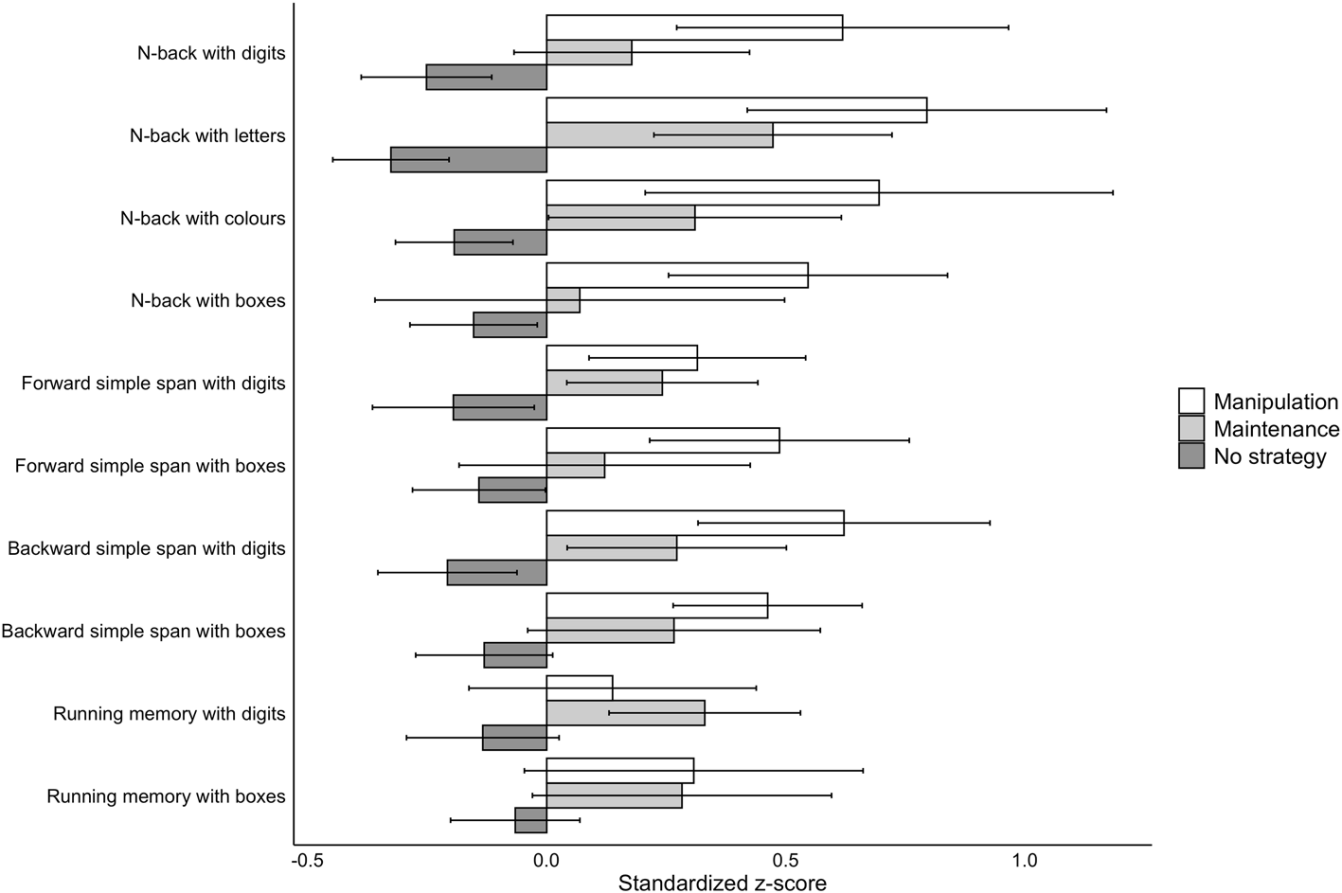
Illustration of task performance grouped by task and strategy use in Experiment 2. Task performance values are standardised using z-scores, providing a comparable metric across different tasks. Whiskers extending from the bars represent 95% confidence intervals. Those participants that had missing data for any of the tasks or whose strategy type for any of the tasks was either other strategy or unspecified past strategy, were excluded from this analysis.

The percentage of participants that used a strategy varied between 18.08 and 41.61%, depending on the task (see Figs. 4, 5; see also Supplementary Information, Appendix F, Tables F.1–F.2 for details). Strategy use was most common in the NBD and FSSD and least common in the RMB. A maintenance strategy (i.e., rehearsal/repetition) was chosen by 7.01–24.91% of the participants. A manipulation strategy (i.e., grouping, updating, or association), on the other hand, was chosen by 10.9–18.77% of the participants.

The number of tasks in which the participants used a strategy varied between 0 and 10. On average, the participants employed a strategy (either maintenance or manipulation) in 5.46 of the 10 tasks (*SD* = 3.81), and the most common number of tasks with strategy employment was three to four. A maintenance strategy was used on average in 2.88 tasks (*SD* = 2.46) and most commonly in four tasks (when used at all). A manipulation strategy was employed on average in 2.58 tasks (*SD* = 1.73) and most commonly in two tasks (when used at all).

#### The second study aim: relationships between strategy use and objective task performance

The participants’ strategy use was examined in relation to their task performance (see Table 3 for details). For this purpose, we ran pairwise Bayesians LMEs for each of the WM tasks. Those participants who employed any strategy (either maintenance or manipulation) performed better than no strategy users in nearly all n-back tasks and both simple span tasks with digits. Maintenance strategy users also displayed better performance on the RMD when compared to the participants who did not use a strategy. On the other hand, there was weak to positive evidence against a performance difference in all box tasks when comparing maintenance strategy users to those who did not use a strategy. Manipulation strategy users also displayed better performance on both simple span tasks with boxes and the NBB when compared to the participants who did not use a strategy. On the other hand, there was weak evidence against a performance difference in the running memory tasks when comparing manipulation strategy users to those who did not use a strategy.

**Table 3:** Parameter estimates for the Bayesian LMEs on strategy sophistication and task performance.

Task	No strategy versus Maintenance		No strategy versus Manipulation		Maintenance versus Manipulation	
	MDiff [95% HDI]^a^	BF ± error %	MDiff [95% HDI]^a^	BF ± error %	MDiff [95% HDI]^b^	BF ± error %
NBD	− 0.17 [− 0.27, − 0.06]	**15.92 ± 0**	− 0.34 [− 0.46, − 0.21]	**> 150 ± 0**	− 0.17 [− 0.33. − 0.01]	1.25 ± 0.02
NBL	− 0.27 [− 0.35, − 0.18]	**> 150 ± 0**	− 0.37 [− 0.48, − 0.27]	**> 150 ± 0**	− 0.1 [− 0.24, 0.04]	1/2.17 ± 0.03
NBC	− 0.18 [− 0.29, − 0.08]	**40.93 ± 0**	− 0.32 [− 0.46, − 0.19]	**> 150 ± 0**	− 0.13 [− 0.32, 0.06]	1/2.22 ± 0.03
NBB	− 0.07 [− 0.21, 0.06]	1/3.45 0** ± **.04	− 0.24 [− 0.35, − 0.14]	**> 150 ± 0**	− 0.16 [− 0.33, 0.02]	1/1.06 ± 0.02
FSSD	− 1.92 [− 3.2, − 0.56]	**6.63 ± 0**	− 2.22 [− 3.71, − 0.81]	**10.8 ± 0**	− 0.31 [− 1.57, 1.04]	1/6.25 ± 0.07
FSSB	− 0.92 [− 2.4, 0.52]	1/2.86 ± 0.04	− 2.26 [− 3.31, − 1.09]	**> 150 ± 0**	-1.24 [− 2.79, 0.31]	1/1.56 ± 0.02
BSSD	− 2.02 [− 3.3, − 0.73]	**12.26 ± 0**	− 3.51 [− 4.94, − 2]	**> 150 ± 0**	-1.42 [− 3, 0.15]	1/1.19 ± 0.02
BSSB	− 1.53 [− 3.22, 0.18]	1/1.32 ± 0.02	− 2.37 [− 3.68, − 1.09]	**60.08 ± 0**	-0.74 [− 2.1, 0.6]	1/2.94 ± 0.03
RMD	− 1.23 [− 2.02, − 0.39]	**9.35 ± 0**	− 0.69 [− 1.74, 0.29]	1/2.78 ± 0.04	0.48 [− 0.38, 1.4]	1/3.33 ± 0.04
RMB	− -1.05 [− 2.51, 0.45]	1/2.08** ± **.02	− 1.18 [− 2.49, 0.04]	1/1.25 ± 0.02	− 0.06 [− 1.55, 1.52]	1/4.55 ± 0.02

Bolded values indicate Bayes Factors (BFs) of 3 or greater. Estimates are from 10 000 samples of the posterior distribution; Mdiff = mean group differences; HDI = highest density interval. NBD = N-back with digits; NBL = N-back with letters; NBC = N-back with colours; FSSD = Forward simple span with digits; FSSB = Forward simple span with boxes; BSSD = Backward simple span with digits; BSSB = Backward simple span with boxes; RMD = Running memory with digits; RMB = Running memory with boxes.

^a^Positive values represent greater performance with no strategy.

^b^Positive values represent greater performance with a maintenance strategy.

When comparing the two strategy sophistication levels, maintenance and manipulation, against each other, there was no positive evidence for a performance difference between users in any of the tasks. Instead, there was either positive evidence against a difference between the users of these two strategies or merely weak evidence for or against a difference.

#### The third study aim: how stimulus type and WM task paradigm modulate spontaneous strategy use

**Figure 6:**
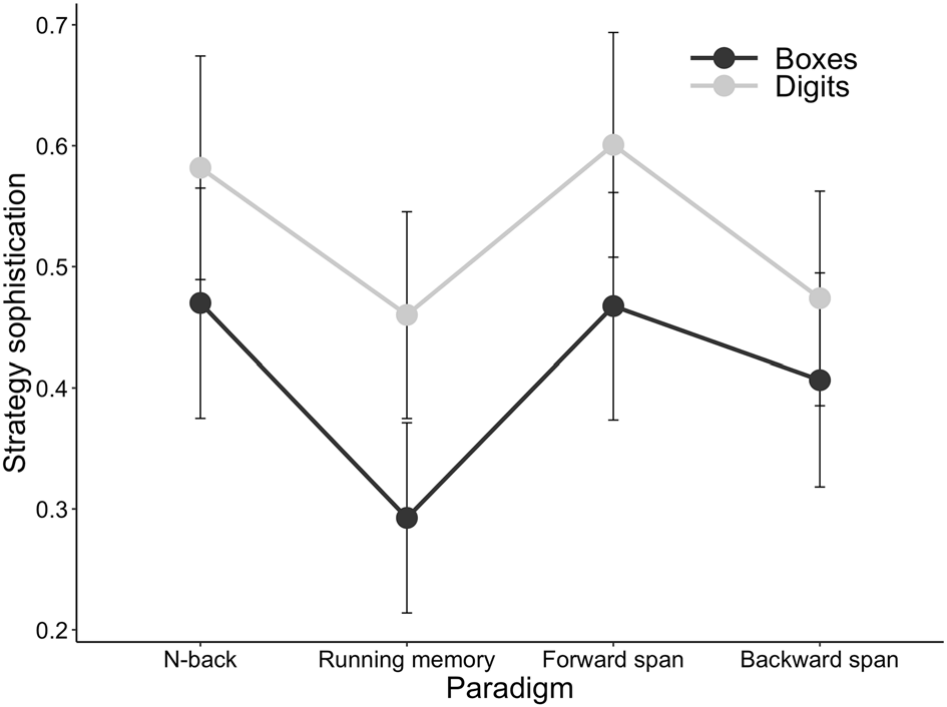
Strategy use by task paradigm and stimulus type in the Bayesian LME analysis in Experiment 2. The y axis displays the mean of strategy sophistication (strategy was treated as an ordinal scale variable with the following values: 0 = No strategy, 1 = Maintenance strategy, 2 = Manipulation strategy). The x-axis depicts the task paradigm.

Next, we examined whether the level of strategy sophistication varied depending on the stimulus type or task paradigm. To utilise the factorial setup available with these data, we specified an LME model with strategy sophistication as the dependent variable, and stimulus type (digits vs. boxes) and paradigm (four task paradigms) as fixed effects. As before, participant served as the random effect. The results revealed very strong evidence for a main effect of stimulus type on strategy sophistication, suggesting that strategy sophistication was higher in tasks with numerical stimuli as compared to their visuospatial counterparts (see Table 4, Fig. 6). Likewise, we observed strong evidence for a main effect of paradigm. A closer inspection of the credible intervals and the pattern of results in Fig. 4 suggests that the paradigm effect is driven by several non-overlapping credible intervals. For instance, n-back elicited higher strategy sophistication than running memory, and forward simple span elicited higher strategy sophistication than backward simple span. We observed very strong evidence against an interaction effect, indicating that stimulus type did not modulate the effect of strategy use in any given task paradigm.

**Table 4:** Parameter estimates for the Bayesian LMEs on strategy sophistication and task characteristics.

Model	Comparison	M_diff_ [95% HDI]^a^	BF_H1_ ± error
Stimulus	Digits versus Boxes	0.13 [0.11, 0.15]	**> 150 ± 1.59%**
Paradigm			**143.72 ± 1.84**%
	N-back versus Running memory	0.13 [0.09, 0.16]	
	N-back versus Forward simple span	− 0.02 [− 0.06, 0.02]	
	N-back versus Backward simple span	0.07 [0.03, 0.11]	
	Running memory versus Forward simple span	− 0.15 [− 0.19, − 0.11	
	Running memory versus Backward simple span	− 0.06 [− 0.10, − 0.02]	
	Forward simple span versus Backward simple span	0.09 [0.05, 0.13]	
Stimulus * Paradigm	Interaction	NA^b^	1/333.33 ± 5.2%

Bolded values indicate Bayes Factors (BFs) of 3 or greater. Estimates are from 10 000 samples of the posterior distribution. Those participants that had missing data or whose strategy type was either other strategy or unspecified past strategy for any of the tasks within a given analysis were excluded from that analysis.

^a^Positive values represent higher strategy sophistication with the first mentioned stimulus type or paradigm.

^b^Too many comparisons.

### Discussion

In Experiment 2 that employed a different constellation of WM tasks compared to Experiment 1, strategy use was clearly less common. For any of the WM tasks, about one third of the participants employed some strategy, although also here the percentage varied strongly between the tasks, as is seen in Figs. 4, 5.

In line with Experiment 1, the results support the association between subjective memory strategy report and objectively measured task performance. As depicted in Table 3, in nine out of ten WM tasks there was positive to very strong evidence for a performance difference in favour of strategy users as compared to the participants who did not use a strategy. The exception was the RMD, where weak evidence for no group differences was observed. However, unlike in Experiment 1, no evidence or at most weak evidence was found for a performance difference between maintenance versus manipulation strategy use.

Importantly, similar to Experiment 1, we found evidence for the effect of stimulus type as well as WM paradigm on strategy sophistication (Table 4). As in Experiment 1, strategy sophistication was overall higher with verbal stimuli (digits) than with nonverbal stimuli (spatial positions). On the other hand, the effect of paradigm on strategy sophistication was different from that observed in Experiment 1. Here, the level of strategy sophistication was higher not only for forward simple span but also for n-back tasks, as compared with backward simple span and running memory.

## General discussion

This study had three aims: to characterise the frequency and types of self-reported mnemonic strategies in common WM tasks, to analyse the associations between strategy use and objective task performance, and to examine how task paradigm and stimulus type affect strategy use. While WM strategy use has been addressed in earlier studies, our knowledge on the three issues listed above is still limited. For example, no previous study that we know of has systematically examined the role of WM task paradigm and stimulus type, and their possible interaction, in strategy use.

The present descriptive results on WM strategy use concur with the view that strategies are a pervasive aspect of human memory performance^4^. Depending on WM task, circa one third (in Experiment 2) up to two-thirds (in Experiment 1) of our participants employed some strategy when facing the tasks for the first time. At the same time, the results reported in Tables 1 and 3 attest to considerable variability in average strategy use between different WM tasks, a phenomenon that pertains to our third research aim discussed below. The frequency of strategy use showed further differences between the experiments, with strategy employment overall being more common in Experiment 1 than in Experiment 2.

A crucial criterion for the validity and usefulness or any subjective memory strategy report is its association with objectively measured task performance. The present results are clear-cut especially when comparing no strategy use with strategy employment, be it maintenance or manipulation. Nearly all WM tasks (eight out of nine in Experiment 1 and nine out of ten in Experiment 2 when considering all relevant contrasts) provided positive to very strong evidence for a performance difference in favour of strategy users as compared to the participants who did not use a strategy. This speaks for the robustness of this effect that has been observed also in previous strategy-related WM studies^6,11,13,15,19^. Morrison et al.^10^ did not find strategy-related performance gains, but they related their null findings to the small number of trials in their WM tasks (5, 6 or 9 stimuli in most tasks). Moreover, evidence for a difference between maintenance versus manipulation was obtained for four WM tasks in Experiment 1, three of which in the expected direction of a better performance when employing a manipulation strategy (cf. also Waris et al.^18^) and one in favour of a maintenance strategy, but for none in Experiment 2. This indicates that the most consistent difference lies between strategy use and no use.

Our test batteries enabled a systematic analysis of the effects of WM task paradigm and stimulus type on strategy sophistication. Utilising factorial designs and Bayesian LMEs, we found consistent evidence for the effect of stimulus type. In most analyses, strategy sophistication was higher with verbal stimuli than with nonverbal stimuli. This result is strengthened by the fact that it stems from two kinds of verbal-nonverbal stimulus contrasts (letters vs. colours; digits vs. visuospatial positions) that represent different participant samples and paradigm constellations. As noted in the Introduction, it has been argued that this content-based division is a fundamental characteristic of WM^45,46^. In WM models such as the multi-component model by Baddeley and Hitch^31^, it is assumed that written letters and digits that we used as our verbal stimuli are automatically recoded into a phonological code^47^. This opens the way to the rich choice of memory strategies for verbal stimuli (for example, grouping stimuli by phonological similarities, creating associations with existing abbreviations, words or with meaningful digit combinations), highlighting the pervasive role of language in complex cognition^36^. In contrast, recent research indicates that colour patches—one of the two types of nonverbal stimuli that we employed—as single-feature stimuli are prone to low-level processing in visual working memory and do not necessarily elicit verbal re-encoding^48^, even though verbal labels exist for them. Likewise, the lack of articulatory suppression effects for the spatial position of an item in visual working memory^49^ speaks against verbal re-encoding of positional information in our WM tasks with boxes. Thus, the lower level of strategy sophistication for our WM tasks with colours or boxes could reflect a relative lack of verbal strategies for these stimuli. A closer look at the primary strategy types (Tables C.1 and F.1 in the Supplementary Information) suggests that perhaps the most consistent difference between verbal versus nonverbal WM task versions concerns the rate of participants who did not use a strategy which is higher for the nonverbal task variants.

In line with Morrison et al.^10^, we found differences in strategy use between task paradigms. However, the effects of paradigm on the level of strategy sophistication differed between the two experiments, indicating that the relationships between these two variables are more complex than between strategy sophistication and stimulus type. The disparate patterns between the experiments might also be due to the differences in the stimuli albeit within the experiments, interactions between paradigm and stimulus type were not found. Nevertheless, there is one consistent finding regarding paradigm effect in both experiments: forward simple span exhibited high rates of strategy sophistication as compared to the other paradigms. A potential reason for this is that forward simple span represents a more familiar and less complex WM task, being relatively easier for implementing a strategy. At the same time, our forward simple span tasks were demanding enough in terms of memory load to trigger strategy use.

The present finding of forward simple span being at the top of strategy implementation amongst the several WM paradigms we examined raises interesting questions as to what actually triggers the spontaneous adoption of memory strategies. Gathercole et al.^50^ recently emphasised the role of task novelty in the development of new cognitive routines (strategies), whereas familiar tasks would be managed by pre-existing routines. In turn, Waris et al.^19^ provided microgenetic evidence that task-initial development of a strategy (not just a pre-existing routine that can be taken directly into use) is not triggered only by task novelty, but can appear also in a familiar, moderately demanding memory task. They note that this echoes in part an earlier proposal that cognitive tasks perceived as moderately difficult—not as easy or exceedingly difficult—are more likely to elicit strategic behaviour^51^. Thus, we can speculate that amongst the present paradigms, the participants may have perceived particularly forward simple span as a moderately difficult task that is rather well-suited for strategy employment.

The present study has some limitations that are worth noting. First, the key strategy variable, strategy sophistication, is based on subjective reports that require verbalisation of a self-generated strategy, which may not always be straightforward for the participant or for the coders. Moreover, in Experiment 2, strategy reports were collected not until after the tasks had been performed, with a potential risk of a memory bias. However, the robust relationships between strategy sophistication and actual performance in both experiments speak for the validity of these introspective reports. Second, the data stem from a web-based study conducted on home computers, and thus we could not control for possible factors in the participants’ surroundings that could have affected their performance. To counteract these potential effects, we conducted a careful screening for background factors that can potentially affect performance, undertook an outlier analysis, and probed for possible cheating. One can also note here that comparisons between lab-based and web-based testing conditions have spoken for the reliability of the results in web-based studies^52–54^. Third, one should point out that the observed relationships between strategy use and objective task performance do not enable causal claims: it is also possible that a better WM leaves more room for strategy generation. However, it is important to point out that studies where participants have received explicit WM strategy instructions indicate subsequent performance improvement when compared with non-instructed controls^6,11,12,15–17,26–28^. Fourth, randomisation of task order, together with the marked variation in strategy use between tasks, prevented us to explore possible sequential effects of spontaneous strategy use. The interesting issue of strategy evolution is most readily studied with setups where the memory task is kept constant and strategy reports are collected after each task block^18,19,55^.

In conclusion, the present results show highly varied self-generated strategy use in WM tasks that is strongly related to task outcomes, systematically modulated by the stimuli (verbal vs. nonverbal) and also affected the type of task paradigm. Spontaneous strategy use should be considered when examining inter- and intra-individual differences in WM performance. The ubiquity of self-generated strategy use calls also for caution in making claims on basic WM storage capacity merely on the basis of WM test scores, as adoption of an effective strategy can have clear-cut effects on the test outcomes.

### Supplementary Information

Supplementary Information.

## Data Availability

The relevant data and analysis code is available at https://osf.io/en6sj.
