# Supplementary material for: Self-reported strategy use in working memory tasks
Source: Sci Rep. 2024 Feb 28;14:4893. doi: 10.1038/s41598-024-54160-3 (PMC10901888; doi:10.1038/s41598-024-54160-3)
Supplement: Supplementary file 1 — Supplementary Information. [file 41598_2024_54160_MOESM1_ESM.docx]

# Supplementary information

## Appendix A: Experiment 1, Strategy coding instructions

The following set of instructions lists the coding rules for the variable strategy type, i.e., strategy classification. The coding rules for another variable, i.e., level of strategy detail, are not included, as this variable is not discussed in this article.

**GENERAL INSTRUCTIONS FOR CODING PARTICIPANTS’ OPEN-ENDED MEMORY STRATEGY DESCRIPTIONS**

- For each strategy description, code the strategy type or types.
  - If the strategy description field is completely empty, do not code anything. (This means that the participant has not completed the task.)
  - If the participant has typed in “None” but nevertheless given a subsequent description of a strategy, evaluate the response from the strategy description.
  - If the participant refers to a strategy s/he used for another task and names that other task, evaluate the response from the strategy description for that other task.
  - If the participant refers to a strategy s/he used for another task that cannot be identified on the basis of the response, mark X as the primary strategy type, leave other strategy types empty, and leave level of detail empty.
  - If the participant reports using any external memory aids (e.g., pen and paper, asking a friend), evaluate the response from the strategy description but also highlight the strategy description in red.
  - Extraneous information not related to strategy use such as “it went bad”, “the task was difficult”, “my mom disturbed me during task performance” should not be taken into account when rating the strategy descriptions, neither comments that merely recount the task characteristics or the task instructions. Keep in mind that strategy is an identifiable, self-generated way or ways of solving a memory task, thus requiring activity from the part of the participant.
- **STRATEGY TYPE CLASSIFICATION:**
  - Mark **strategy type 1** for the strategy that is mentioned first in the description. There can only be one primary strategy.
  - If there are no additional strategies, leave the other strategy type fields empty.
  - If there are two strategies, mark the strategy that is mentioned second as **strategy type 2**.
  - If needed, continue in the same way up to four strategy types, always coding the strategy types in the order they are mentioned.
  - If the participant lists more than four strategy types, the additional strategy types are not coded.

| **CODING CRITERIA: STRATEGY TYPE CLASSIFICATION** | | |
| --- | --- | --- |
| **Code** | **Type** | **Examples** |
| **0** | **No strategy use**   - No strategy - No clear evidence of strategy use - Repetition of instructions - Response unrelated to strategy use, comments on understanding instructions, or comments on task difficulty - Guessing, intuition, instinct, familiarity, ‘gut feeling’ | - “No”, “None” - “I pressed the N-key if the current colour was the same as the colour presented before it, or the M-key if it was not the same” - “I chose the items that felt most familiar”, “I just entered some random words” - “I did not try to actively memorise the words, but rather 'went with the flow' and noted each word as it appeared” - “This task was difficult”, “I did not understand what I was supposed to do” - Only “Yes” or “I used a strategy” or “Attempted to use a mnemonic” (with no description as to what the strategy/mnemonics would have been) - “I memorised all the items” |
| **1** | **Rehearsal / Repetition**   - Rehearsing items verbally, nonverbally or visuomotorically - *E.g. repeating, counting out loud, speaking in head, saying in mind, yelling the items, rehearsing quietly, counting with fingers, pointing at with cursor, following items with mouse* | - “I repeated the colours” - “I rehearsed the items in my mind” - “Counted out the letters”, “I repeated all the colours aloud” - “Counted the digits with my fingers”, “I followed each colour with the mouse” |
| **2** | **Grouping**   - Grouping items together - *E.g. chunking, pairing, memorizing in groups, remembering in chunks, thinking in pairs, dividing into subsets, subdividing into sets, splitting into shorter sequences* | - “I memorised the colours in chunks” - “I grouped the letters in pairs” - “When the sequence was long enough I remembered the digits in groups of 4” - “I split the items into different series, and compared those to each other” (also other (comparison)) |
| **3** | **Updating**   - Updating items - *E.g. replacing, substituting, switching items, dropping old items along the way when new ones appear* | - “I tried to replace each colour one at a time as a new colour came up” - “I dropped the first digit when a new one appeared” - “Substituted the first letter with the latest letter that appeared on the screen” - “Tried to remember the first set n of colours and then replaced out the old for the new ones.” (strategy 1 = grouping) - “Throughout the sequence, I tried to keep the last 4 in my head, updating them as the sequence progressed.” (strategy 1 = grouping) |
| **4** | **Association**   - Linking items with some mental representations (visual, spatial, verbal) in mind - *E.g. visualizing items as objects, forming spatial images, visualizing items in locations, creating associations with words or syllables that rhyme with the items, forming meaningful units such as words or sentences, making a story of the items* | - “I imagined each item as an object”, “I tried to visualise the letters as snakes”, “I tried to associate each digit with some image in my mind” - “I imagined each item in a familiar location”, “I tried to visualise the digits as chess positions”, “I tried to associate each letter with some place on a map” - “I tried to make syllables out of the letters”, “I used rhyming to keep the items in my mind” - “I connected items that, for me, share a meaningful connection”, “I thought of furniture to help recall the words chair, table, and shelf”, “I exchanged the colours to corresponding words (RED, BLUE)” - “I created words from the letters (e.g., C-R-S = Corn – Rose – Sand)”, “I created a sentence out of the stimuli” - “I used the items to create a story”, “Made up a story based on the words to remember them better” |
| **5** | **Selective focus**   - Focusing on only some of the items | - “I focused on remembering only a subset of the items and disregarded the rest” - “Just concentrated on the easy ones as the task was quite difficult” - “Focused on the ones I had previously forgotten” - “Fixated only on the first 4 digits” |
| **6** | **Other strategy** | - “I made up a song based on the letters” - “I compared the new items with the old items”, “I tried to match them to those I saw previously” - “I visualised the numbers”, “I tried to visualise the letter sequence in my mind” (these do not count as associations as they fail to show evidence for a link with some mental representations in mind) |
| **999** | **Unspecified past strategy**   - Referring to a strategy used in a previous task that cannot be identified on the basis of the response | - “Same”, “Same strategy” - “I used the same strategy as in the previous task”, “I used the same strategy as before” |

## Appendix B: Experiment 1. Details on data preprocessing

| **Table B.1** | | | | | |
| --- | --- | --- | --- | --- | --- |
| *Summary table of the reasons for excluding participants from the strategy type analyses* | | | | | |
|  |  |  |  |  |  |
| Task | Missing data | Colour-blindness | Unreliable effort | Total number  of *n* excluded | *N* after  exclusions |
| NBD | 2 | 0 | 3 | 5 | 195 |
| NBL | 0 | 0 | 3 | 3 | 197 |
| NBC | 0 | 5 | 2 | 7 | 193 |
| FSSL | 0 | 0 | 0 | 0 | 200 |
| FSSC | 0 | 5 | 0 | 5 | 195 |
| RML | 1 | 0 | 0 | 1 | 199 |
| RMC | 0 | 5 | 0 | 5 | 195 |
| SUD | 0 | 0 | 0 | 0 | 200 |
| SUC | 0 | 5 | 0 | 5 | 195 |
| *Note.* NBD = N-back with digits; NBL = N-back with letters; NBC = N-back with colours; FSSL = Forward simple span with letters; FSSC = Forward simple span with colours; RML = Running memory with letters; RMC = Running memory with colours; SUD = Selective updating of digits; SUC = Selective updating of colours. | | | | | |

| **Table B.2** | | | | | | | |
| --- | --- | --- | --- | --- | --- | --- | --- |
| *Summary table of the reasons for excluding participants from the strategy class analyses* | | | | | | | |
|  |  |  |  |  |  |  |  |
| Task | Missing data | Colour-blindness | Unreliable effort | Missing strategy | Other or unspecified strategy | Total number of *n* excluded | *N* after exclusions |
| NBD | 2 | 0 | 3 | 0 | 9 | 14 | 186 |
| NBL | 0 | 0 | 3 | 0 | 6 | 9 | 191 |
| NBC | 0 | 5 | 2 | 0 | 14 | 21 | 179 |
| FSSL | 0 | 0 | 0 | 0 | 3 | 3 | 197 |
| FSSC | 0 | 5 | 0 | 0 | 0 | 5 | 195 |
| RML | 1 | 0 | 0 | 0 | 4 | 5 | 195 |
| RMC | 0 | 5 | 0 | 0 | 3 | 8 | 192 |
| SUD | 0 | 0 | 0 | 0 | 7 | 7 | 193 |
| SUC | 0 | 5 | 0 | 0 | 9 | 14 | 186 |
| *Note.* NBD = N-back with digits; NBL = N-back with letters; NBC = N-back with colours; FSSL = Forward simple span with letters; FSSC = Forward simple span with colours; RML = Running memory with letters; RMC = Running memory with colours; SUD = Selective updating of digits; SUC = Selective updating of colours. | | | | | | | |

## Appendix C: Experiment 1. Details on strategy use

| **Table C.1** | | | |
| --- | --- | --- | --- |
| *Number (*n*) and percentage (%) of participants using different strategies to complete the cognitive tasks* | | | |
|  |  |  |  |
| Task | Strategy type | *n* | Percentage (%) |
| NBD | No strategy | 84 | 43.08 |
|  | Rehearsal/Repetition | 73 | 37.44 |
|  | Grouping | 23 | 11.79 |
|  | Other strategy | 7 | 3.59 |
|  | Selective focus | 4 | 2.05 |
|  | Updating | 2 | 1.03 |
|  | Unknown past strategy | 2 | 1.03 |
|  | Association | 0 | 0.00 |
|  | *Total* | *195* | *100* |
| NBL | No strategy | 98 | 49.75 |
|  | Rehearsal/Repetition | 75 | 38.07 |
|  | Grouping | 12 | 6.09 |
|  | Other strategy | 5 | 2.54 |
|  | Selective focus | 3 | 1.52 |
|  | Association | 2 | 1.02 |
|  | Updating | 1 | 0.51 |
|  | Unknown past strategy | 1 | 0.51 |
|  | *Total* | *197* | *100* |
| NBC | No strategy | 101 | 52.33 |
|  | Rehearsal/Repetition | 63 | 32.64 |
|  | Other strategy | 13 | 6.74 |
|  | Grouping | 11 | 5.70 |
|  | Updating | 2 | 1.04 |
|  | Association | 1 | 0.52 |
|  | Selective focus | 1 | 0.52 |
|  | Unknown past strategy | 1 | 0.52 |
|  | *Total* | *193* | *100* |
| FSSL | Rehearsal/Repetition | 66 | 33.00 |
|  | Association | 55 | 27.50 |
|  | No strategy | 51 | 25.50 |
|  | Grouping | 23 | 11.50 |
|  | Other strategy | 3 | 1.50 |
|  | Selective focus | 2 | 1.00 |
|  | Updating | 0 | 0.00 |
|  | Unknown past strategy | 0 | 0.00 |
|  | *Total* | *200* | *100* |
| FSSC | Rehearsal/Repetition | 93 | 47.69 |
|  | No strategy | 68 | 34.87 |
|  | Association | 15 | 7.69 |
|  | Selective focus | 10 | 5.13 |
|  | Grouping | 9 | 4.62 |
|  | Updating | 0 | 0.00 |
|  | Other strategy | 0 | 0.00 |
|  | Unknown past strategy | 0 | 0.00 |
|  | *Total* | *195* | *100* |
| RML | No strategy | 74 | 37.19 |
|  | Rehearsal/Repetition | 60 | 30.15 |
|  | Association | 34 | 17.09 |
|  | Grouping | 14 | 7.04 |
|  | Selective focus | 13 | 6.53 |
|  | Other strategy | 2 | 1.01 |
|  | Unknown past strategy | 2 | 1.01 |
|  | Updating | 0 | 0.00 |
|  | *Total* | *199* | *100* |
| RMC | No strategy | 83 | 42.56 |
|  | Rehearsal/Repetition | 71 | 36.41 |
|  | Grouping | 14 | 7.18 |
|  | Selective focus | 13 | 6.67 |
|  | Association | 8 | 4.10 |
|  | Updating | 3 | 1.54 |
|  | Other strategy | 3 | 1.54 |
|  | Unknown past strategy | 0 | 0.00 |
|  | *Total* | *195* | *100* |
| SUD | Rehearsal/Repetition | 90 | 45.00 |
|  | No strategy | 82 | 41.00 |
|  | Selective focus | 9 | 4.50 |
|  | Grouping | 7 | 3.50 |
|  | Other strategy | 6 | 3.00 |
|  | Updating | 3 | 1.50 |
|  | Association | 2 | 1.00 |
|  | Unknown past strategy | 1 | 0.50 |
|  | *Total* | *200* | *100* |
| SUC | Rehearsal/Repetition | 95 | 48.72 |
|  | No strategy | 75 | 38.46 |
|  | Selective focus | 8 | 4.10 |
|  | Other strategy | 8 | 4.10 |
|  | Updating | 4 | 2.05 |
|  | Association | 3 | 1.54 |
|  | Grouping | 1 | 0.51 |
|  | Unknown past strategy | 1 | 0.51 |
|  | *Total* | *195* | *100* |
| *Note.* NBD = N-back with digits; NBL = N-back with letters; NBC = N-back with colours; FSSL = Forward simple span with letters; FSSC = Forward simple span with colours; RML = Running memory with letters; RMC = Running memory with colours; SUD = Selective updating of digits; SUC = Selective updating of colours. | | | |

| **Table C.2** |  |  |  |  |  |  |
| --- | --- | --- | --- | --- | --- | --- |
| *Number (n) and percentage (%) of participants using different strategies to complete the cognitive tasks, and objective task performance for each task.* | | | | | |  |
|  |  |  |  |  |  |  |
|  |  |  |  |  |  |  |
| Task | Strategy sophistication | *n* | Percentage of participants (%) | Task performance *M* | Task performance *SD* |  |
| NBD | Manipulation | 29 | 15.59 | 3.39 | 0.89 |  |
|  | Maintenance | 73 | 39.25 | 2.84 | 1.01 |  |
|  | No strategy | 84 | 45.16 | 2.27 | 0.67 |  |
|  | *Total* | *186* | *100* | *2.67* | *0.94* |  |
| NBL | Manipulation | 18 | 9.42 | 3.25 | 0.78 |  |
|  | Maintenance | 75 | 39.27 | 2.67 | 0.88 |  |
|  | No strategy | 98 | 51.31 | 2.21 | 0.64 |  |
|  | *Total* | *191* | *100* | *2.49* | *0.82* |  |
| NBC | Manipulation | 15 | 8.38 | 3.41 | 0.51 |  |
|  | Maintenance | 63 | 35.20 | 2.72 | 0.77 |  |
|  | No strategy | 101 | 56.42 | 2.29 | 0.63 |  |
|  | *Total* | *179* | *100* | *2.54* | *0.75* |  |
| FSSL | Manipulation | 80 | 40.61 | 27.30 | 6.97 |  |
|  | Maintenance | 66 | 33.50 | 26.45 | 6.69 |  |
|  | No strategy | 51 | 25.89 | 21.41 | 9.19 |  |
|  | *Total* | *197* | *100* | *25.49* | *7.73* |  |
| FSSC | Manipulation | 34 | 17.44 | 24.74 | 11.07 |  |
|  | Maintenance | 93 | 47.69 | 26.92 | 8.02 |  |
|  | No strategy | 68 | 34.87 | 23.65 | 10.92 |  |
|  | *Total* | *195* | *100* | *25.40* | *9.74* |  |
| RML | Manipulation | 61 | 31.28 | 21.03 | 4.76 |  |
|  | Maintenance | 60 | 30.77 | 20.38 | 4.70 |  |
|  | No strategy | 74 | 37.95 | 18.18 | 5.98 |  |
|  | *Total* | *195* | *100* | *19.75* | *5.36* |  |
| RMC | Manipulation | 38 | 19.79 | 21.29 | 4.75 |  |
|  | Maintenance | 71 | 36.98 | 18.85 | 5.55 |  |
|  | No strategy | 83 | 43.23 | 17.30 | 6.60 |  |
|  | *Total* | *192* | *100* | *18.66* | *6.05* |  |
| SUD | Manipulation | 21 | 10.88 | 26.57 | 8.84 |  |
|  | Maintenance | 90 | 46.63 | 34.51 | 8.32 |  |
|  | No strategy | 82 | 42.49 | 26.21 | 8.64 |  |
|  | *Total* | *193* | *100* | *30.12* | *9.41* |  |
| SUC | Manipulation | 16 | 8.60 | 30.31 | 7.07 |  |
|  | Maintenance | 95 | 51.08 | 33.92 | 8,68 |  |
|  | No strategy | 75 | 40.32 | 26.13 | 11,30 |  |
|  | *Total* | *186* | *100* | *30.747* | *10,35* |  |
|  |  |  |  |  |  |  |

*Note.* Those participants whose strategy type was either other strategy or unspecified past strategy for the respective task were excluded from this analysis. NBD = N-back with digits; NBL = N-back with letters; NBC = N-back with colours; FSSL = Forward simple span with letters; FSSC = Forward simple span with colours; RML = Running memory with letters; RMC = Running memory with colours; SUD = Selective updating of digits; SUC = Selective updating of colours.

## Appendix D: Experiment 2, Strategy coding instructions

The following set of instructions lists the coding rules for the variable strategy type, i.e., strategy classification. The coding rules for other variables, i.e., level of strategy detail or the number of strategies, are not included, as these variables are not discussed in this article. The original instructions were in Swedish, this is a translation.

**GENERAL INSTRUCTIONS**

- If the participant clicked on ”No” in response to the question ” Did you use any strategies when performing this task?” but despite this wrote something in the response field, base your scoring on the comment instead of the “No” response.
- If the participant clicked on ”Yes” in response to the question ” Did you use any strategies when performing this task?” but did not write anything in the response field, score as “Other strategy”.
- If the participant clicked on ”Yes in response to the question ” Did you use any strategies when performing this task?” but wrote something that is not classifiable as a strategy (e.g. “This task was difficult”), in the response field, score as “other strategy”.
- If the participant refers to a previous response or to some of their previous responses, score as the description they are referring to.
  - Exception 1: If e.g. the participant writes a detailed strategy description for task 1 but also writes ”Same as previous” for task 2 and task 3, score task 2 and 3 based on the description for task 1.
  - Exception 2: If the participant writes a detailed description for task 1 and a detailed description for task 2 and ”Same as previous” for task 3, score task 3 based on the description for task 2.

| **CODING CRITERIA: STRATEGY CLASSIFICATION** | |
| --- | --- |
| Attention! If the participant reports more than one strategy in their comment, always base your scoring on the strategy that is named first | |
| **Scoring** | **Example** |
| **Rehearsal (1)** | - “I repeated the digits silently in my mind” - “I repeated a list of letters in my mind” - “Repeating out loud the letters” |
| **Visuo-motor rehearsal (2)** | - “followed the boxes with the mouse” - “Touched the white boxes on the screen with my fingers” - “I used my computer mouse to follow the boxes that appeared on the screen” - “I counted the digits with my fingers” |
| **Grouping (3)** | - “I created groups of 3 digits” - “I grouped the letters in pairs” - “When the sequence was long enough I remembered the items in groups of 4” - “I remembered the digits in groups” |
| **Updating (4)** | - “I created a group of digits in my mind and dropped the last digit when a new digit appeared” - “Replaced the first letter with the latest letter that appeared on the screen.” - “I tried to replace each color one at a time as a new color came up” |
| **Grouping and comparison (5)** | - “I split the digits into different series, and compared those to each other” - “Held each sequence of digits in mind, removed the first and added a new digit to the end. After that, I checked if it was the same as the one just dropped.” |
| **Semantics (6)** | - “I created words from the letters (e.g., C-R-S = Corn – Rose – Sand)” - “I converted the digits to melodies, e.g., 1356 = DO-MI-SO-LA” |
| **Phonology (7)** | - “I made up lists based on first syllables of the digits” - “I tried to make syllables out of the letters” |
| **Imagery (8)** | - “I tried to associate each digit with some image in my mind” - “I tried to visualise the letters as snakes” |
| **Visualization (9)** | - “I visualized the numbers” - “I tried memorise the locations of the boxes visually” - “I tried to visualise the letter sequence in my mind” |
| **Familiarity (10)** | - “I chose the letters that felt most familiar” - “I recalled the digits that were familiar” |
| **Guessing (11)** | - “I just used intuition” - “I started somewhere in the middle of the sequence, and did not memorise the first digits in the sequence at all” |
| **Other strategies (12)**  *"Liberal" take, that is also e.g. a “Yes” in the response field is classified as “Other”* | - “I made up a song based on the letters” - “Yes” - “I tried to keep all the digits in my mind” - “This task was difficult” |
| **No strategy use (13)**  *”Strict” take, that is if one only says ”No”, repeats the test instructions or describes something that is not related to strategy use* | - “I pressed the N-key if the current white box was the same as the white box presented before it, or the M-key if it was not the same. - “No” |

## Appendix E: Experiment 2. Details on data preprocessing

| **Table E.1** | | | | | |
| --- | --- | --- | --- | --- | --- |
| *Summary table of the reasons for excluding participants from the strategy type analyses* | | | | | |
|  | | | | | |
| Task | Missing data | Colour blindness | Unreliable effort | Total number  of *n* excluded | *N* after exclusions |
| NBD | 1 | 0 | 8 | 9 | 281 |
| NBL | 3 | 0 | 5 | 8 | 282 |
| NBC | 3 | 2 | 5 | 10 | 280 |
| NBB | 2 | 0 | 11 | 13 | 277 |
| FSSD | 2 | 0 | 0 | 2 | 288 |
| FSSB | 0 | 0 | 5 | 5 | 285 |
| BSSD | 2 | 0 | 0 | 2 | 288 |
| BSSB | 0 | 0 | 3 | 3 | 287 |
| RMD | 0 | 0 | 1 | 1 | 289 |
| RMB | 0 | 0 | 0 | 0 | 290 |
| *Note.* NBD = N-back with digits; NBL = N-back with letters; NBC = N-back with colours; FSSD = Forward simple span with digits; FSSB = Forward simple span with boxes; BSSD = Backward simple span with digits; BSSB = Backward simple span with boxes; RMD = Running memory with digits; RMB = Running memory with boxes. | | | | | |

| **Table E.2** | | | | | | | |
| --- | --- | --- | --- | --- | --- | --- | --- |
| *Summary table of the reasons for excluding participants from the strategy class analyses* | | | | | | | |
|  |  |  |  |  |  |  |  |
| Task | Missing data | Colour blindness | Unreliable effort | Missing strategy | Other strategy | Total number of *n* excluded | *N* after exclusions |
| NBD | 1 | 0 | 8 | 0 | 16 | 25 | 265 |
| NBL | 3 | 0 | 5 | 0 | 10 | 18 | 272 |
| NBC | 3 | 2 | 5 | 3 | 11 | 24 | 266 |
| NBB | 2 | 0 | 11 | 3 | 6 | 22 | 268 |
| FSSD | 2 | 0 | 0 | 0 | 14 | 16 | 274 |
| FSSB | 0 | 0 | 5 | 0 | 8 | 13 | 277 |
| BSSD | 2 | 0 | 0 | 0 | 19 | 21 | 269 |
| BSSB | 0 | 0 | 3 | 1 | 6 | 10 | 280 |
| RMD | 0 | 0 | 1 | 1 | 23 | 25 | 265 |
| RMB | 0 | 0 | 0 | 2 | 17 | 19 | 271 |
| *Note.* NBD = N-back with digits; NBL = N-back with letters; NBC = N-back with colours; FSSD = Forward simple span with digits; FSSB = Forward simple span with boxes; BSSD = Backward simple span with digits; BSSB = Backward simple span with boxes; RMD = Running memory with digits; RMB = Running memory with boxes. | | | | | | | |

## Appendix F: Experiment 2. Details on strategy use

| **Table F.1** | | | |
| --- | --- | --- | --- |
| *Number (*n*) and percentage (%) of participants using different strategies to complete the cognitive tasks* | | | |
|  |  |  |  |
| Task | Strategy type | n | Percentage (%) |
| NBD | No strategy | 155 | 55.16 |
|  | Rehearsal | 63 | 22.42 |
|  | Grouping | 27 | 9.61 |
|  | Other strategies | 16 | 5.69 |
|  | Grouping and comparison | 7 | 2.49 |
|  | Updating | 6 | 2.14 |
|  | Visualization | 3 | 1.07 |
|  | Visuo-motor rehearsal | 2 | 0.71 |
|  | Familiarity | 1 | 0.36 |
|  | Transformation | 1 | 0.36 |
|  | Semantics | 0 | 0.00 |
|  | Phonology | 0 | 0.00 |
|  | Imagery | 0 | 0.00 |
|  | Guessing | 0 | 0.00 |
|  | *Total* | *281* | *100* |
| NBL | No strategy | 176 | 62.41 |
|  | Rehearsal | 59 | 20.92 |
|  | Grouping | 14 | 4.96 |
|  | Other strategies | 9 | 3.19 |
|  | Semantics | 7 | 2.48 |
|  | Grouping and comparison | 5 | 1.77 |
|  | Visualization | 5 | 1.77 |
|  | Updating | 4 | 1.42 |
|  | Phonology | 2 | 0.71 |
|  | Guessing | 1 | 0.35 |
|  | Visuo-motor rehearsal | 0 | 0.00 |
|  | Imagery | 0 | 0.00 |
|  | Familiarity | 0 | 0.00 |
|  | Transformation | 0 | 0.00 |
|  | *Total* | *282* | *100* |
| NBC | No strategy | 186 | 67.15 |
|  | Rehearsal | 49 | 17.69 |
|  | Grouping | 10 | 3.61 |
|  | Visualization | 10 | 3.61 |
|  | Other strategies | 9 | 3.25 |
|  | Grouping and comparison | 4 | 1.44 |
|  | Updating | 3 | 1.08 |
|  | Familiarity | 2 | 0.72 |
|  | Guessing | 2 | 0.72 |
|  | Transformation | 2 | 0.72 |
|  | Visuo-motor rehearsal | 0 | 0.00 |
|  | Semantics | 0 | 0.00 |
|  | Phonology | 0 | 0.00 |
|  | Imagery | 0 | 0.00 |
|  | *Total* | *277* | *100* |
| NBB | No strategy | 191 | 69.71 |
|  | Visualization | 30 | 10.95 |
|  | Visuo-motor rehearsal | 22 | 8.03 |
|  | Transformation | 8 | 2.92 |
|  | Grouping | 6 | 2.19 |
|  | Rehearsal | 5 | 1.82 |
|  | Other strategies | 5 | 1.82 |
|  | Imagery | 4 | 1.46 |
|  | Grouping and comparison | 2 | 0.73 |
|  | Guessing | 1 | 0.36 |
|  | Updating | 0 | 0.00 |
|  | Semantics | 0 | 0.00 |
|  | Phonology | 0 | 0.00 |
|  | Familiarity | 0 | 0.00 |
|  | *Total* | *274* | *100* |
| FSSD | No strategy | 160 | 55.56 |
|  | Rehearsal | 64 | 22.22 |
|  | Grouping | 48 | 16.67 |
|  | Other strategies | 14 | 4.86 |
|  | Grouping and comparison | 1 | 0.35 |
|  | Visualization | 1 | 0.35 |
|  | Visuo-motor rehearsal | 0 | 0.00 |
|  | Updating | 0 | 0.00 |
|  | Semantics | 0 | 0.00 |
|  | Phonology | 0 | 0.00 |
|  | Imagery | 0 | 0.00 |
|  | Guessing | 0 | 0.00 |
|  | Familiarity | 0 | 0.00 |
|  | Transformation | 0 | 0.00 |
|  | *Total* | *288* | *100* |
| FSSB | No strategy | 200 | 70.18 |
|  | Visualization | 29 | 10.18 |
|  | Visuo-motor rehearsal | 23 | 8.07 |
|  | Transformation | 12 | 4.21 |
|  | Other strategies | 8 | 2.81 |
|  | Grouping | 6 | 2.11 |
|  | Imagery | 5 | 1.75 |
|  | Rehearsal | 2 | 0.7 |
|  | Updating | 0 | 0.00 |
|  | Grouping and comparison | 0 | 0.00 |
|  | Semantics | 0 | 0.00 |
|  | Phonology | 0 | 0.00 |
|  | Familiarity | 0 | 0.00 |
|  | Guessing | 0 | 0.00 |
|  | *Total* | *285* | *100* |
| BSSD | No strategy | 181 | 62.85 |
|  | Rehearsal | 49 | 17.01 |
|  | Grouping | 37 | 12.85 |
|  | Other strategies | 18 | 6.25 |
|  | Updating | 1 | 0.35 |
|  | Visualization | 1 | 0.35 |
|  | Guessing | 1 | 0.35 |
|  | Visuo-motor rehearsal | 0 | 0.00 |
|  | Grouping and comparison | 0 | 0.00 |
|  | Semantics | 0 | 0.00 |
|  | Phonology | 0 | 0.00 |
|  | Imagery | 0 | 0.00 |
|  | Familiarity | 0 | 0.00 |
|  | Transformation | 0 | 0.00 |
|  | *Total* | *288* | *100* |
| BSSB | No strategy | 210 | 73.43 |
|  | Visualization | 24 | 8.39 |
|  | Visuo-motor rehearsal | 22 | 7.69 |
|  | Transformation | 9 | 3.15 |
|  | Grouping | 7 | 2.45 |
|  | Other strategies | 6 | 2.1 |
|  | Imagery | 5 | 1.75 |
|  | Rehearsal | 3 | 1.05 |
|  | Updating | 0 | 0.00 |
|  | Grouping and comparison | 0 | 0.00 |
|  | Semantics | 0 | 0.00 |
|  | Phonology | 0 | 0.00 |
|  | Familiarity | 0 | 0.00 |
|  | Guessing | 0 | 0.00 |
|  | *Total* | *286* | *100* |
| RMD | No strategy | 175 | 60.76 |
|  | Rehearsal | 57 | 19.79 |
|  | Grouping | 25 | 8.68 |
|  | Other strategies | 21 | 7.29 |
|  | Updating | 6 | 2.08 |
|  | Visualization | 2 | 0.69 |
|  | Guessing | 2 | 0.69 |
|  | Visuo-motor rehearsal | 0 | 0.00 |
|  | Grouping and comparison | 0 | 0.00 |
|  | Semantics | 0 | 0.00 |
|  | Phonology | 0 | 0.00 |
|  | Imagery | 0 | 0.00 |
|  | Familiarity | 0 | 0.00 |
|  | Transformation | 0 | 0.00 |
|  | *Total* | *288* | *100* |
| RMB | No strategy | 222 | 77.08 |
|  | Visualization | 17 | 5.9 |
|  | Other strategies | 16 | 5.56 |
|  | Visuo-motor rehearsal | 15 | 5.21 |
|  | Transformation | 8 | 2.78 |
|  | Rehearsal | 4 | 1.39 |
|  | Grouping | 3 | 1.04 |
|  | Updating | 1 | 0.35 |
|  | Imagery | 1 | 0.35 |
|  | Guessing | 1 | 0.35 |
|  | Grouping and comparison | 0 | 0.00 |
|  | Semantics | 0 | 0.00 |
|  | Phonology | 0 | 0.00 |
|  | Familiarity | 0 | 0.00 |
|  | *Total* | *288* | *100* |
| *Note.* NBD = N-back with digits; NBL = N-back with letters; NBC = N-back with colours; FSSD = Forward simple span with digits; FSSB = Forward simple span with boxes; BSSD = Backward simple span with digits; BSSB = Backward simple span with boxes; RMD = Running memory with digits; RMB = Running memory with boxes. | | | |

| **Table F.2** |  |  |  |  |  |  |
| --- | --- | --- | --- | --- | --- | --- |
| *Number (n) and percentage (%) of participants using different strategies to complete the cognitive tasks, and objective task performance for each task* | | | | | |  |
|  |  |  |  |  |  |  |
| Task | Strategy sophistication | *n* | Percentage of participants (%) | Task performance  *M* | Task performance  *SD* |  |
|  |  |  |  |  |  |  |
| NBD | Manipulation | 44 | 16.6 | 3.02 | 0.92 |  |
|  | Maintenance | 66 | 24.91 | 2.67 | 0.81 |  |
|  | No strategy | 155 | 58.49 | 2.32 | 0.69 |  |
|  | *Total* | *265* | *100* | *2.53* | *0.81* |  |
| NBL | Manipulation | 37 | 13.6 | 2.89 | 0.77 |  |
|  | Maintenance | 59 | 21.69 | 2.67 | 0.65 |  |
|  | No strategy | 176 | 64.71 | 2.13 | 0.56 |  |
|  | *Total* | *272* | *100* | *2.35* | *0.68* |  |
| NBC | Manipulation | 29 | 10.9 | 3.00 | 0.96 |  |
|  | Maintenance | 51 | 19.17 | 2.71 | 0.81 |  |
|  | No strategy | 186 | 69.92 | 2.34 | 0.63 |  |
|  | *Total* | *266* | *100* | *2.48* | *0.75* |  |
| NBB | Manipulation | 50 | 18.66 | 2.79 | 0.74 |  |
|  | Maintenance | 27 | 10.07 | 2.44 | 0.78 |  |
|  | No strategy | 191 | 71.27 | 2.28 | 0.67 |  |
|  | *Total* | *268* | *100* | *2.39* | *0.72* |  |
| FSSD | Manipulation | 50 | 18.25 | 34.74 | 7.20 |  |
|  | Maintenance | 64 | 23.36 | 34.08 | 7.22 |  |
|  | No strategy | 160 | 58.39 | 30.13 | 9.79 |  |
|  | *Total* | *274* | *100* | *31.89* | *9.03* |  |
| FSSB | Manipulation | 52 | 18.77 | 29.52 | 7.25 |  |
|  | Maintenance | 25 | 9.03 | 26.80 | 5.48 |  |
|  | No strategy | 200 | 72.2 | 24.85 | 7.41 |  |
|  | *Total* | *277* | *100* | *25.90* | *7.44* |  |
| BSSD | Manipulation | 39 | 14.5 | 30.69 | 8.24 |  |
|  | Maintenance | 49 | 18.22 | 27.63 | 6.98 |  |
|  | No strategy | 181 | 67.29 | 23.44 | 8.68 |  |
|  | *Total* | *269* | *100* | *25.25* | *8.75* |  |
| BSSB | Manipulation | 45 | 16.07 | 30.62 | 5.45 |  |
|  | Maintenance | 25 | 8.93 | 29.00 | 6.14 |  |
|  | No strategy | 210 | 75 | 25.71 | 8.72 |  |
|  | *Total* | *280* | *100* | *26.80* | *8.28* |  |
| RMD | Manipulation | 33 | 12.45 | 20.79 | 4.64 |  |
|  | Maintenance | 57 | 21.51 | 21.84 | 4.13 |  |
|  | No strategy | 175 | 66.04 | 19.30 | 5.85 |  |
|  | *Total* | *265* | *100* | *20.03* | *5.47* |  |
| RMB | Manipulation | 30 | 11.07 | 16.90 | 6.29 |  |
|  | Maintenance | 19 | 7.01 | 16.74 | 4.31 |  |
|  | No strategy | 222 | 81.92 | 14.42 | 6.78 |  |
|  | *Total* | *271* | *100* | *14.86* | *6.63* |  |

*Note.* Those participants whose strategy type was either other strategy or unspecified past strategy for the respective task were excluded from this analysis. NBD = N-back with digits; NBL = N-back with letters; NBC = N-back with colours; FSSD = Forward simple span with digits; FSSB = Forward simple span with boxes; BSSD = Backward simple span with digits; BSSB = Backward simple span with boxes; RMD = Running memory with digits; RMB = Running memory with boxes.
